# Two Arabidopsis Chloroplast GrpE Homologues Exhibit Distinct Biological Activities and Can Form Homo- and Hetero-Oligomers

**DOI:** 10.3389/fpls.2019.01719

**Published:** 2020-01-22

**Authors:** Pai-Hsiang Su, Hsuan-Yu Lin, Yen-Hsun Lai

**Affiliations:** ^1^ Agricultural Biotechnology Research Center, Academia Sinica, Taipei, Taiwan; ^2^ Biotechnology Center in Southern Taiwan, Academia Sinica, Tainan, Taiwan

**Keywords:** chloroplast GrpE homologue (CGE), embryo lethal, Hsp70, DnaK, luciferase refolding assay, oligomerization

## Abstract

Flowering plants have evolved two distinct clades of chloroplast GrpE homologues (CGEs), which are the nucleotide exchange factor for Hsp70. In Arabidopsis, they are named AtCGE1 (At5g17710) and AtCGE2 (At1g36390). Characterization of their corresponding T-DNA insertion mutants revealed that there is no visible change in phenotype except a defect in protein import in an *AtCGE2*-knockout mutant under normal growth conditions. However, the embryo development of an *AtCGE1*-knockout mutant was arrested early at the globular stage. An *AtCGE1*-knockdown mutant, harboring a T-DNA insertion in the 5′-UTR region, exhibited growth retardation and protein import defect, and its mutant phenotypes became more severe when *AtCGE2* was further knocked out. Sub-organellar distribution implied that AtCGE2 might be important for membrane biology due to its preferential association with chloroplast membranes. Biochemical studies and complementation tests showed that only AtCGE1, but not AtCGE2, can effectively rescue the heat-sensitive phenotype of *Escherichia coli grpE* mutant and robustly stimulate the refolding of denatured luciferase by DnaK. Interestingly, AtCGE1 and AtCGE2 are tending to form heterocomplexes, which exhibit comparable co-chaperone activity to AtCGE1 homocomplexes. Our data indicate that AtCGE1 is the principle functional homologue of GrpE. The possibility that AtCGE2 has a subsidiary or regulatory function through homo- and/or hetero-oligomerization is discussed.

## Introduction

Almost all living organisms have 70-kD heat shock proteins (Hsp70) in most cellular compartments, including the cytoplasm, endoplasmic reticulum (ER), mitochondria, and chloroplasts. In addition to the general function of assisting protein folding, emerging roles of Hsp70 are being reported in animal cells, including gene regulation, protein translocation and degradation, signal transduction, and cell apoptosis (Mayer and Bukau, 2005; Joly et al., 2010; Kampinga and Craig, 2010). However, experimental evidence to support the biological functions of Hsp70 in the plant system is still scarce. Sung and Guy (2003) showed that the cytosolic Hsp70 overexpression can enhance the heat tolerance of Arabidopsis under certain conditions. Leborgne-Castel et al. (1999) reported that overexpressing ER-localized Hsp70 (Bip) in tobacco plants alleviated ER stress and increased drought tolerance, but no effect on heat tolerance was observed. In green algae, it has been shown that chloroplast Hsp70 may be involved in the protection of the photosystem and repair after photoinhibition (Schroda et al., 1999; Yokthongwattana et al., 2001). In *Chlamydomonas*, chloroplast Hsp70 mediates the assembly of vesicle inducing protein in plastid 1 (VIPP1), which may facilitate the biogenesis and maintenance of thylakoid membrane (Liu et al., 2007). However, whether chloroplast Hsp70 in flowering plants also exhibits similar functions to its algal counterparts is still uncertain. Recently it has been demonstrated that plastid Hsp70s are essential for chloroplast and plant development and are important for seed basal thermotolerance in Arabidopsis (Su and Li, 2008; Latijnhouwers et al., 2010). Genetic and biochemical analyses revealed that plastid Hsp70s function as the molecular motor driving precursor proteins into chloroplasts in moss, pea, and Arabidopsis (Shi and Theg, 2010; Su and Li, 2010; Liu et al., 2014).

The chloroplast Hsp70 chaperone system is derived from the cyanobacterial endosymbiotic ancestor. In general the system is composed of three core members: Hsp70, DnaJ, and GrpE homologues. In Arabidopsis, there are two chloroplast Hsp70s (cpHsc70-1 and cpHsc70-2), two chloroplast GrpE homologues (AtCGE1 and AtCGE2), and 19 chloroplast DnaJ homologues (Su and Li, 2008; Hu et al., 2012; Chiu et al., 2013). The functions of individual co-chaperone family members are still largely unknown. It has been shown that small J proteins (AtJ8, AtJ11, and AtJ20) may contribute to the optimization of photosynthesis (Chen et al., 2010). In *Chlamydomonas*, CGE1 cooperates with Hsp70 to mediate the assembly of VIPP1 protein complex (Liu et al., 2007) and the formation of Hsp70/Hsp90 multichaperone complex (Willmund et al., 2008). *Physcomitrella* CGE proteins play a crucial role in protein import (Shi and Theg, 2010). In recent, de Luna-Valdez et al. (2019) proposed that land plants have evolved two independent groups of CGE proteins with distinguishable variations in conserved short motifs. It is suggested that AtCGE1 is involved in specific physiological phenomena in Arabidopsis, such as the chloroplast response to heat stress, and the correct oligomerization of photosynthesis-related LHCII complex (de Luna-Valdez et al., 2019). However, the physiological significance of AtCGE2 and the difference in co-chaperone activities between AtCGE1 and AtCGE2 are still unknown.

From genomic survey and phylogenetic analysis, we revealed that flowering plants have evolved two distinct clades of CGE homologues prior to the divergence of monocot and dicot lineages. To understand the functional differences between these two clades of CGEs in flowering plants, we performed genetic and biochemical analyses of the two Arabidopsis CGEs. Our data show that two AtCGEs exhibit different co-chaperone activities. AtCGE1 functions as a bona fide GrpE homologue with an essential function in embryo development, and AtCGE2 seems to be subsidiary or have a regulatory function to diversify the CGE co-chaperone activities.

## Materials and Methods

### Data Mining and Phylogenetic Analysis

Genomic resources were obtained from National Center for Biotechnology Information (NCBI), Ensembl_Plants, the DOE Joint Genome Institute, and the Rice Genome Annotation Project through the web sites listed in **Table S1**. Sequences which were ambiguous due to poor sequencing data were not used for further analysis. Finally, a total of 62 CGE protein sequences from 34 sequenced genomes were adopted for the construction of a phylogenetic tree by ClustalW alignment and the neighbor-joining method in MEGA6 software (Tamura et al., 2013).

### Plant Growth Conditions

For plate culture, Arabidopsis seeds were sterilized with 1.5% sodium hyperchloride for 10 min, washed with sterile water 5 times, and plated on 0.3% gellan gum-solidified 1× Murashige and Skoog (MS) medium containing 2% sucrose. After a 3-d cold stratification, seeds were grown in a growth chamber under 16-h photoperiod with a light intensity around 70 μmol m^−2^ s^−1^ at 22°C. For soil culture, Arabidopsis seeds were imbibed and cold-stratified for 3 d in a refrigerator and sowed on a 9:1:1 mixture of peat, vermiculite, and perlite under a 16-h photoperiod with a light intensity approximately 100 μmol m^−2^ s^−1^ at 24°C.

### Identification and Characterization of the T-DNA Insertion Mutants of *CGE*s

The candidate T-DNA insertion mutants were searched by the SIGnAL T-DNA Express platform (http://signal.salk.edu/cgi-bin/tdnaexpress). Seeds of candidate lines, FLAG_136H03 (*atcge2-1*), FLAG_079G12 (*atcge1-3*), SALK_004126 (*atcge2-2*), SALK_005391 (*atcge1-2*), and WiscDxLoxHs045_03B (*atcge1-1*) were obtained from ABRC (Arabidopsis Biological Resource Center) and INRA (French National Institute for Agricultural Research) (Brunaud et al., 2002; Alonso et al., 2003; Woody et al., 2007) and confirmed by genomic PCR with specific primers. For amplifying the wild-type copy of *AtCGE2*, primer pairs of CGE2P-S and CGE2t-AS were used. Primer pairs of CGE2P-S and Tag5 were used for amplifying the *atcge2-1* specific T-DNA copy, and primer pairs of LBa1-2 and CGE2t-AS were used for identifying the *atcge2-2*. For amplifying the wild-type copy of *AtCGE1*, primer pairs of CGE1P-S + CGE1I2-AS or CGE1E1-S + CGE1t-AS were used. Primer pairs of CGE1P-S and pDs-LoxHs-L4' were used for amplifying the *atcge1-1* specific T-DNA copy; primer pairs of LBa1-2 and CGE2t-AS were used for identifying the *atcge1-2*; and primer pairs of CGE1P-S and Tag5 were used for identifying the *atcge1-3*. The T-DNA insertion sites were verified by sequencing the PCR products. Primers pairs used for checking *AtCGE2* and *AtCGE1* transcripts by reverse transcription-polymerase chain reaction (RT-PCR) were CGE2-S + CGE2-AS and CGE1E1-S + CGE1-AS, respectively. Individual insertion mutants were back crossed to their relative wild type to select their single insertion mutants for phenotype characterization, crossing, and functional analyses. Total chlorophyll was determined by the method described by Lichtenthaler (Lichtenthaler, 1987). Sequences of oligonucleotide primers are listed in **Table S2**.

### *In Vitro* Translation and Protein Import Assay

[^35^S]-Methione-labeled prRBCS were *in vitro* transcribed/translated with TNT^®^ Coupled Wheat Germ Extract System driven by SP6 promoter (Promega). Chloroplasts were isolated from 24-d-old seedlings grown on MS medium containing 2% sucrose. Import assays were conducted as described in Perry et al. (1991), except the grinding buffer was modified to 50 mM HEPES-KOH (pH 8.0), 330 mM sorbitol, 2 mM EDTA, and 0.5% bovine serum albumin. After import, intact chloroplasts were re-isolated through 40% Percoll cushion for SDS-PAGE analysis (NuPAGE 4–12% Bis-Tris gel, Invitrogen), and import was visualized by radiography with intensifying screens or by phosphor-imaging. Quantification of gel bands was performed using the Typhoon Trio phosphor-imager and ImageQuant TL software (GE Healthcare).

### Sub-Organellar Fractionation

Intact Arabidopsis chloroplasts were isolated from 21-d-old plate-grown seedlings of wild type, and suspended in import buffer. Lysis of chloroplasts was performed by resuspending pelleted intact chloroplasts in hypotonic buffer [50 mM HEPES-KOH (pH 8.0), 50 mM NaCl, and 5 mM MgCl_2_], or in alkaline extraction buffer containing 0.1 M Na_2_CO_3_ (pH 11.5). Lysis mixture was incubated at 4°C for 30 min with mild vortex and then frozen overnight in −20°C freezer. Thereafter, the thawed samples were separated into membranes and soluble fractions by ultracentrifugation at 100,000 g for 45 min, and repeated once to ensure a sufficient fractionation. Total chloroplast protein, lysed supernatant, and pellet (membrane fraction) were then resolved on PAGE, transferred onto polyvinylidene fluoride (PVDF) membrane, and decorated with Western blotting against AtCGE2 and AtCGE1 antibodies, respectively. The CBR-stained LHCB and RBCS were used as the controls of membrane and stromal fraction, respectively.

### Expression of Recombinant CGE Proteins and Antibody Preparation

Using proofreading Phusion DNA polymerase, the coding sequences of mature regions of *AtCGE2* and *AtCGE1* were amplified by PCR with specific primer pairs, CGE2-NdeI-S + CGE2-XhoI-AS and CGE1-NdeI-S + CGE1-XhoI-AS, respectively. The amplified DNA fragments were cut with *Nde*I and *Xho*I, and subcloned onto pET22b (Novagen) expression vectors trimmed with the same restriction enzymes to generate the 6xHis-tagged recombinant constructs, designated as pET22b-AtCGE2 and pET22b-AtCGE1, respectively. After being confirmed by DNA sequencing, the recombinant plasmids were transformed into *Escherichia coli*. BL21 (DE3) for IPTG (Isopropyl β-D-1-thiogalactopyranoside) induced overexpression. Recombinant AtCGE proteins were affinity-purified by Talon beads (Clontech Laboratories) for chaperone activity assay and customer antibody production (GeneTex). To generate the V294A mutation of AtCGE1 on pET22b, site-directed mutagenesis was conducted with mutated primer pairs, CGE1-V294A-S + CGE1-V294A-AS. To express AtCGE1/2 heterodimer, the coding sequence of mature AtCGE1 was amplified by PCR with primer pairs XhoI-CGE1-S and XhoI-CGE1-AS, and then subcloned onto *Xho*I-cut pET22b-Ptac-AtCGE2 plasmid (see below) to generate a polycistronic cassette named as pET22b-Ptac-AtCGE2-CGE1, in which AtCGE1 is tagged with 6xHis and AtCGE2 was tag-free. Purification procedure of mutated AtCGE1 and AtCGE1/2 heterodimer was was according to the user's manual of Talon metal affinity resins (Clontech Laboratories).

### Functional Complementation of *E. coli grpE* Mutant

The heat-sensitive *E. coli* DA16 harbors a *grpE280* mutation (Johnson et al., 1989). Because DA16 lacks the λDE3 fragment for inducible expression of T7 RNA polymerase, the expression cassette driven by T7 promoter on the pET22b vector could not be turned on by IPTG. Therefore, we replaced the T7 promoter with *tac* promoter to generate the pET22b-Ptac vector, which enables IPTG-induced overexpression in DA16. Then the coding sequences of *grpE* and the mature regions of *AtCGE2* and *AtCGE1* were subcloned onto this vector as described above. The resulting plasmids were named as pET22b-Ptac-GrpE, -AtCGE2, and -AtCGE1, respectively. For complementation test, wild-type *grpE* construct tagged with 6xHis was used as a positive control. After 2-h induction by a serial concentration of IPTG at 30°C on solid agar plates, the transformed bacteria were heat challenged at high temperature as indicated. The overnight cultured plates were photographed by digital camera to record the results of complementation. To co-express both AtCGEs in DA16 mutant, the T7 promoter of pCOLA vector (Novagen) was replaced by *tac* promoter to generate pCOLA-Ptac vector, then the coding sequence of *AtCGE2* was subcloned on this vector to generate pCOLA-Ptac-AtCGE2 plasmid with S-tag in frame. Finally, pCOLA-Ptac-AtCGE2 and pET22b-Ptac-AtCGE1 were co-transformed into *E. coli* DA16.

### Luciferase Refolding Assay

Using the pJKE7 plasmid (Takara Bio), which harbors *dnaK*, *dnaJ*, and *grpE* genes, as template DNA, the protein-coding sequences of *dnaK*, *dnaJ*, and *grpE* were amplified by PCR, and were respectively subcloned onto the expression vectors of pACYC-T7, pET21d, and pET22b-Ptac. Their resulting plasmids were named pACYC-T7-DnaK, pET21d-DnaJ, and pET22b-Ptac-GrpE and were transformed into *E. coli* for overexpression. All recombinant proteins of DnaK, DnaJ, GrpE, and AtCGEs were fused with a 6xHis tag and were affinity purified by Talon metal affinity beads according to the user's manual (Clontech Laboratories). The *E. coli* BL21(DE3) strain was used for IPTG-induced overexpression of DnaJ, GrpE, and AtCGE proteins, and the KRX strain (Promega) was used for rhamnose-controlled overexpression of DnaK. Purified proteins underwent dialysis in common buffer [25 mM Hepes-KOH (pH 7.8), 5 mM MgCl_2_, and 5 mM DTT] containing 100 mM NaCl and were quantified by BCA protein assay kit (Pierce), then stored in −80°C deepfreezer at 100 μM concentration in small aliquot. Refolding of denatured firefly luciferase was modified from Sugimoto et al. (2007). Briefly, luciferase was chemically denatured in 6 M guanidine hydrochloride for 30 min at 25°C. Refolding was conducted by adding denatured luciferase into common buffer containing 100 mM KCl, chaperone mixture, and 3 mM ATP, mixing well, and incubating for 30 min at 25°C. The protein concentrations used are DnaK (5 μM), DnaJ (1 μM), and luciferase (100 nM). For GrpE and AtCGEs, serial concentrations from 0.5 to 32 μM were used as indicated in the figures. After reaction, 10 μl of refolding mixture was sampled for luciferase activity assay in a final 125 μl common buffer containing 1 mM ATP and 0.25 mM D-luciferin. The luminescence was measured by a luminometer (Victor X2 multilabel plate reader, PerkinElmer). Activity of native luciferase was assayed as a positive control.

### Crosslinking of Purified AtCGEs by Glutaraldehyde

Crosslinking experiment was modified from Wu et al. (1996). Five micromolar proteins in common buffer were crosslinked with glutaraldehyde at 0.05, 0.1, and 0.2% for 10 min. After crosslinking, their oligomeric states were visualized by PAGE analysis and CBR staining.

## Results

### Phylogenetic Analysis Revealed That Flowering Plants Have Evolved Two Distinct Clades of CGEs

**Figure 1:**
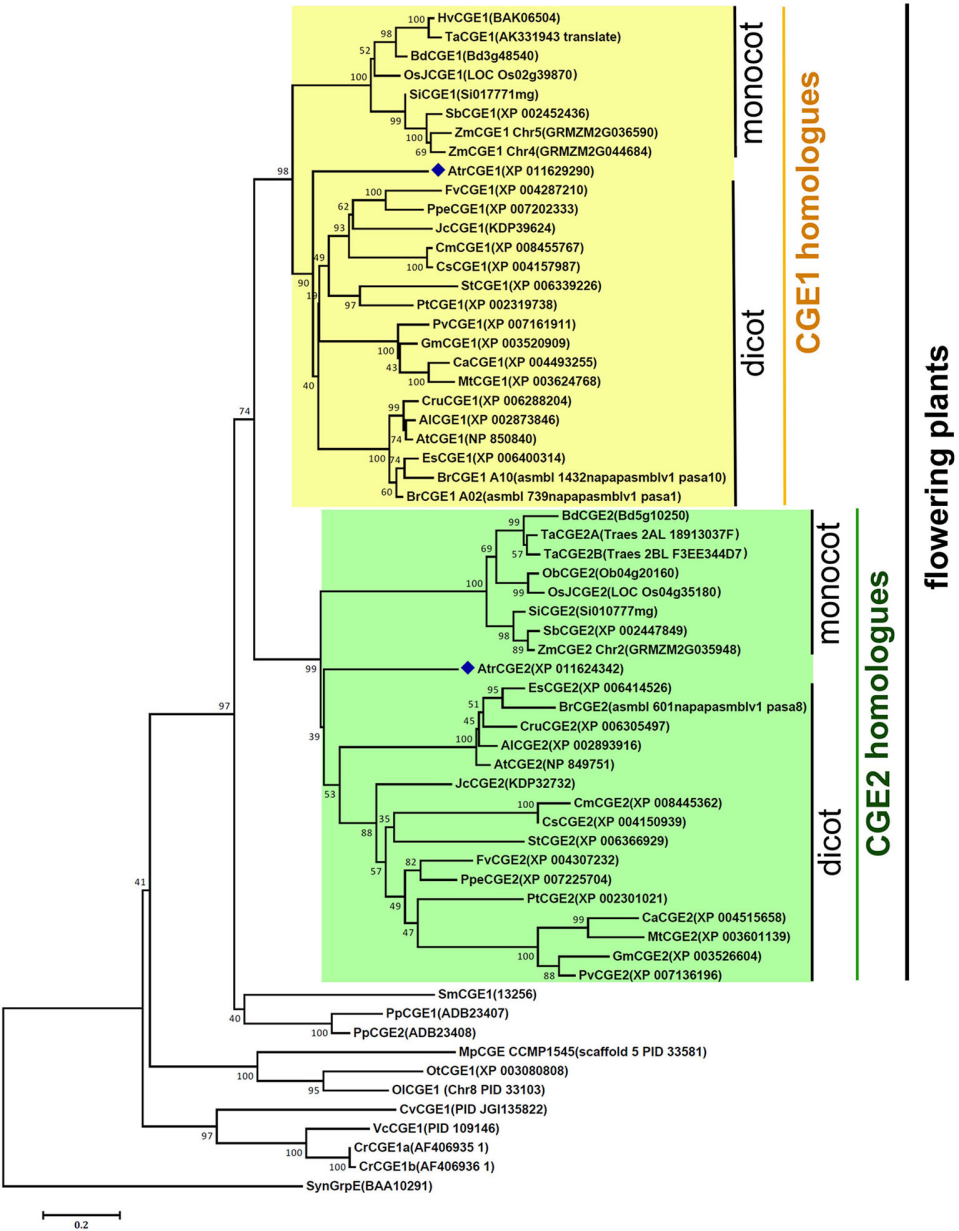
A phylogenetic tree of species CGE proteins. The tree was constructed by using the neighbor-joining method in MEGA6 (19) with 62 CGE proteins (see aligned sequences in **Data S1**). Bootstrap Values are shown as a percentage of 1,000 replicates. The evolutionary distances were computed using the Dayhoff matrix based method and are in the units of the number of amino acid substitutions per site. The rate variation among sites was modeled with a gamma distribution (shape parameter = 2). All ambiguous positions were removed for each sequence pair. Al, *Arabidopsis lyrata*; At, *Arabidopsis thaliana*; Atr, *Amborella trichopoda*; Bd, *Brachypodium distachyon*; Br, *Brassica rapa*; Ca, *Cicer arietinum*; Cm, *Cucumis melo*; Cr, *Chlamydomonas reinhardtii*; Cru, *Capsella rubella*; Cs, Cucumis sativus; Cv, *Chlorella variabilis*; Es, *Eutrema salsugineum*; Fv, *Fragaria vesca*; Gm, *Glycine max*; Hv, *Hordeum vulgare*; Jc, *Jatropha curcas*; Mp, *Micromonas pusilla*; Mt, *Medicago truncatula*; Ob, *Oryza brachyantha*; OsJ, *Oryza sativa* Japonica Group; Ol, *Ostreococcus lucimarinus*; Ot, *Ostreococcus tauri*; Pp, *Physcomitrella patens*; Ppe, *Prunus persica*; Pt, *Populus trichocarpa*; Pv, *Phaseolus vulgaris*; Sb, *Sorghum bicolor*; Si, *Setaria italica*; Sm, *Selaginella moellendorffii*; St, *Solanum tuberosum*; Syn, *Synechocystis*; Ta, *Triticum aestivum*; Vc, *Volvox carteri*; Zm, *Zea mays*.

Survey of sequenced plant genomes revealed that most flowering plants contain at least two copies of *CGE* homologues, instead of a single genomic copy as found in cyanobacteria, algae, and *Selaginella*. To understand their evolutionary relationship, a phylogenetic tree was constructed with 62 CGE protein sequences from 34 sequenced genomes using the MEGA6 program (Tamura et al., 2013). As shown in **Figure 1**, flowering plants have evolved two distinct clades of CGEs, which were grouped into CGE1 and CGE2 homologues, respectively, with a bootstrap value (74% of 1,000 replicates). It was shown that two CGEs of *Amborella*, which is an ancient plant diverging near the base of flowering plant lineages, were also grouped into CGE1 and CGE2 clades respectively, indicating that these two CGE clades of flowering plants may have evolved prior to the divergence of monocot and dicot lineages. There are duplicated copies in the same clade in some species with more recent whole genome duplication or polyploidy, such as *Brassica rapa*, *Triticum aestivum*, and *Zea mays*. Notably, *Physcomitrella patens* also has two CGEs, which seem to be derived from a more recent duplication based on their high similarity with 85% sequence identity in mature proteins, and are grouped into the same clade. In *Chlamydomonas*, two CGE isoforms (namely CGE1a and CGE1b) with only a two-amino acid difference are encoded by a single gene with an alternative splicing of mRNA (Schroda et al., 2001).

### Characterizations of Arabidopsis *atcge1* and *atcge2* Mutants

**Figure 2:**
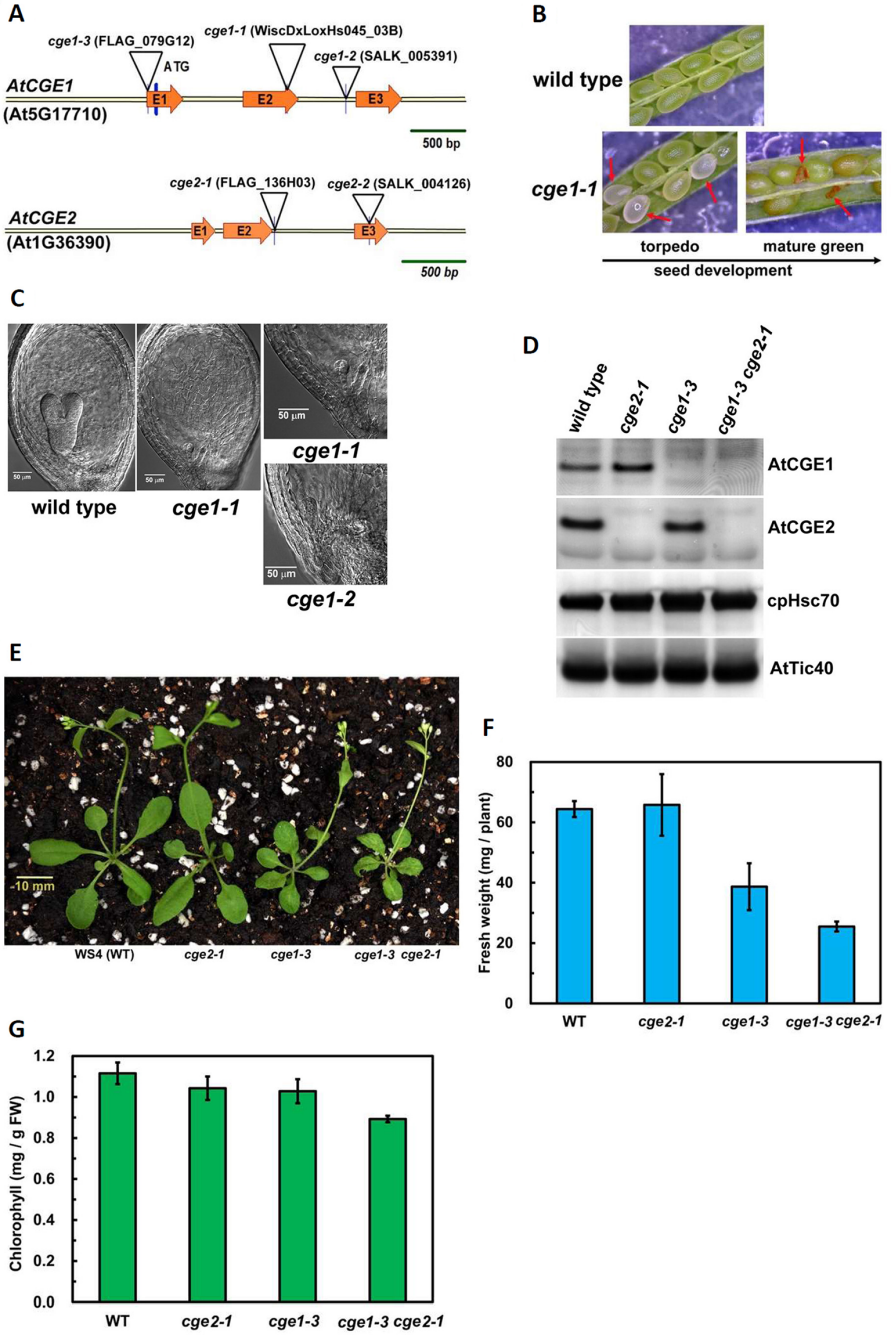
Characterization of *atcge* mutants. **(A)** Illustration of T-DNA insertion sites in the genomic fragments of *AtCGE1* (At5g17710) and *AtCGE2* (At1g36390). Both AtCGE1 and AtCGE2 contain three exons, designated as E1, E2, and E3. **(B)** Observing aborted seeds of *atcge1-1* mutant in the siliques at the torpedo and the mature green stages under dissecting microscopy. **(C)** Microscopic observation of embryogenesis in wild type and *atcge* mutants at the torpedo stage. **(D)** Western blot of total chloroplast proteins isolated from wild type and *atcge* mutants. Samples were loaded with equal chlorophyll. Antibodies used are indicated on the right sides of blots. **(E)** Photograph of 21-d-old soil-grown seedlings. Fresh weight **(F)** and chlorophyll content **(G)** of the above-ground part of wild type and *atcge* mutants. Data are the means ± SD of three replicates.

To understand the biological significance of having two clades of CGE homologues, we conducted further studies using Arabidopsis CGEs. Data mining of a microarray database revealed that there is not a large difference in RNA expression patterns and levels between *AtCGE2* and *AtCGE1* (access through Arabidopsis eFP browser at http://bar.utoronto.ca/efp_arabidopsis/cgi-bin/efpWeb.cgi), implying possible functional redundancy. *AtCGE* T-DNA insertion mutants were further isolated for characterization (**Figure 2** and **Figure S1**). The actual T-DNA insertion sites after being re-confirmed by genomic PCR and DNA sequencing are illustrated in **Figure 2A**. Knockout mutations of *AtCGE1* (*atcge1-1* and *atcge1-2*) caused an embryo lethal phenotype. During seed development, approximately one quarter of the seeds produced from the heterozygous *atcge1-1* mutant showed bleaching color at the torpedo stage and brown aborted seeds at the mature green stage (**Figure 2B**). Differential interference contrast (DIC) microscopic observation of developing seeds at the torpedo stage revealed that embryogenesis of these bleaching seeds was arrested at the early globular stage (**Figure 2C**). It is surprising that *AtCGE2* cannot compensate the defect of *AtCGE1* although their RNA expression patterns and levels are quite similar, suggesting that the two proteins may have different functions or activities. We also isolated a viable *AtCGE1*-knockdown mutant, *atcge1-3*, which has a T-DNA insertion in the 5′-UTR region and produces almost no detectable AtCGE1 protein (**Figure 2D**). To avoid the uncertain effect caused by different ecotypes, *atcge1-3* and *atcge2-1* (both are of WS4 ecotype) were selected for further analysis. There was no visible mutant phenotype in *AtCGE2*-knockout plants (*atcge2-1*) under normal growth conditions (**Figure 2E**). The *atcge1-3* mutant exhibited a growth retardation phenotype (**Figure 2E**). In 21-d soil-grown seedlings, the above-ground tissue fresh weight of *atcge1-3* only reached 60% of that of the wild type (**Figure 2F**). When *atcge1-3* was crossed to *atcge2-1*, their double mutant progeny showed more severe growth retardation (**Figures 2E, F**). The fresh weight and chlorophyll content of *atcge1-3 atcge2-1* were reduced to 40% and 80% of that of the wild type, respectively (**Figures 2F, G**). Taken together these results show that AtCGE1 is essential for embryogenesis, and important for plant growth. In contrast, AtCGE2 is dispensable under normal growth conditions, but becomes important when AtCGE1 is knocked down. AtCGE2 may be partially functionally redundant to AtCGE1 or may mediate AtCGE1 function under some conditions.

### The *atcge* Mutants Defect in Chloroplast Protein Import

**Figure 3:**
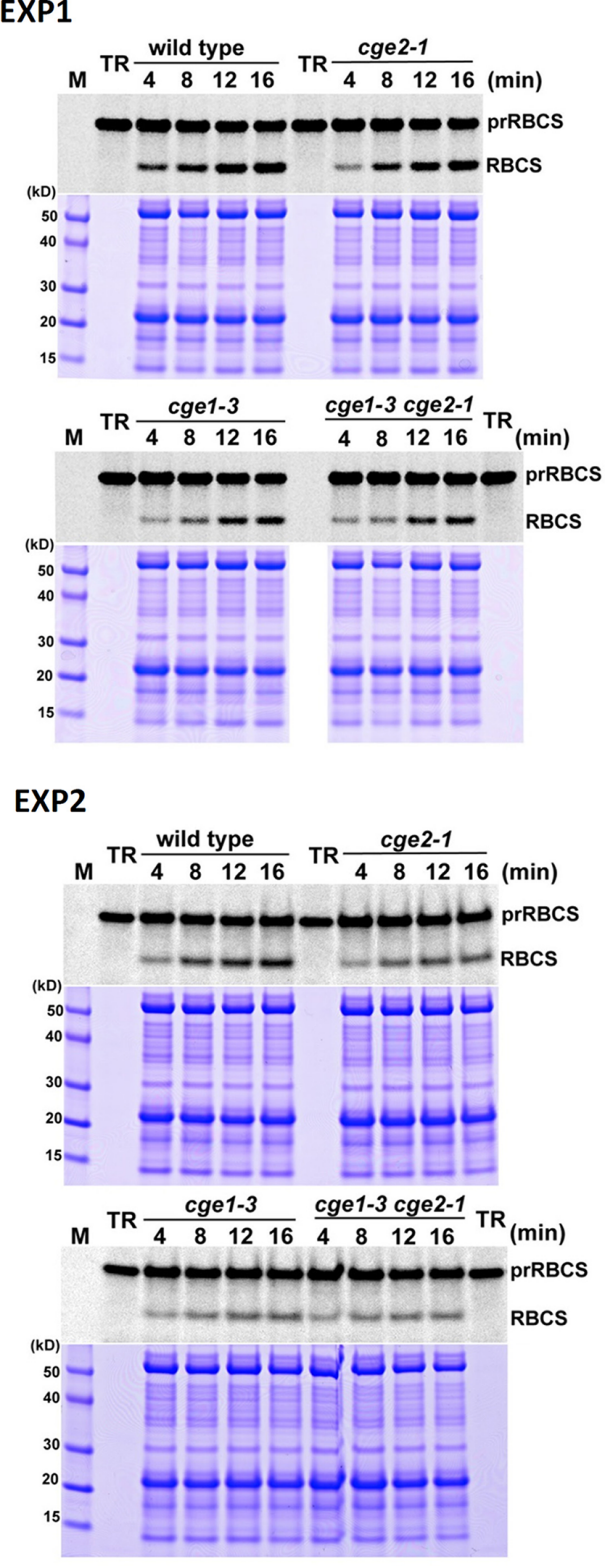
The *atcge* mutants had defects in chloroplast protein import. The prRBCS import into chloroplasts isolated from wild-type and mutant seedlings grown on MS medium for 24 d. *In vitro* import assays were conducted for 4 to 16 min with [^35^S]Methionine-labeled prRBCS. After import, intact chloroplasts were reisolated and analyzed by PAGE. The gels were stained with Coomassie blue, scanned, and then dried for phosphoimaging. The region of each stained gel between the endogenous large and small subunits of Rubisco is shown below the phosphoimage. Two independent experiments (EXP1 and EXP2) are shown. TR, *in vitro*–translated [^35^S]Met-prRBCS before the import reactions.

It has been shown that chloroplast Hsp70 is engaged in chloroplast protein import in *Physcomitrella*, Arabidopsis, and pea (Shi and Theg, 2010; Su and Li, 2010), and *Physcomitrella* CGEs are important for efficient protein import (Shi and Theg, 2010). To investigate whether flowering plant CGEs are also involved in this essential process, *in vitro* protein import of [^35^S]-methione-labeled precursor to the small subunit of Rubisco (prRBCS) was assayed. The *atcge1-3* chloroplasts showed a severe import defect, and their imported RBCS only reached to about a half of that of wild-type chloroplasts after a 16-min import (**Figure 3** and **Figure S2**). It was noticeable that *atcge2-1* chloroplasts also exhibited a remarkable import defect, but to a quite milder degree compared to *atcge1-3* (**Figure 3** and **Figure S2**), although the *atcge2-1* plant did not show visible mutant appearance under normal growth conditions (**Figure 2E**). The *atcge1-3 atcge2-1* double mutant seemed to display a more severe import defect than both single mutants (**Figure 3** and **Figure S2**). These results indicated that AtCGE2 might still be important for plant vigor under normal conditions. It is noted that the steady-state level of AtCGE1 protein seems to be increased in *atcge2-1* mutant (**Figure 2D**), possibly implying the loss of AtCGE2 may have a consequential effect on plant physiology. In summary, both AtCGEs may contribute to chloroplast protein import by mediating the action of chloroplast Hsp70s.

### Sub-Organellar Fractionation of AtCGEs

**Figure 4:**
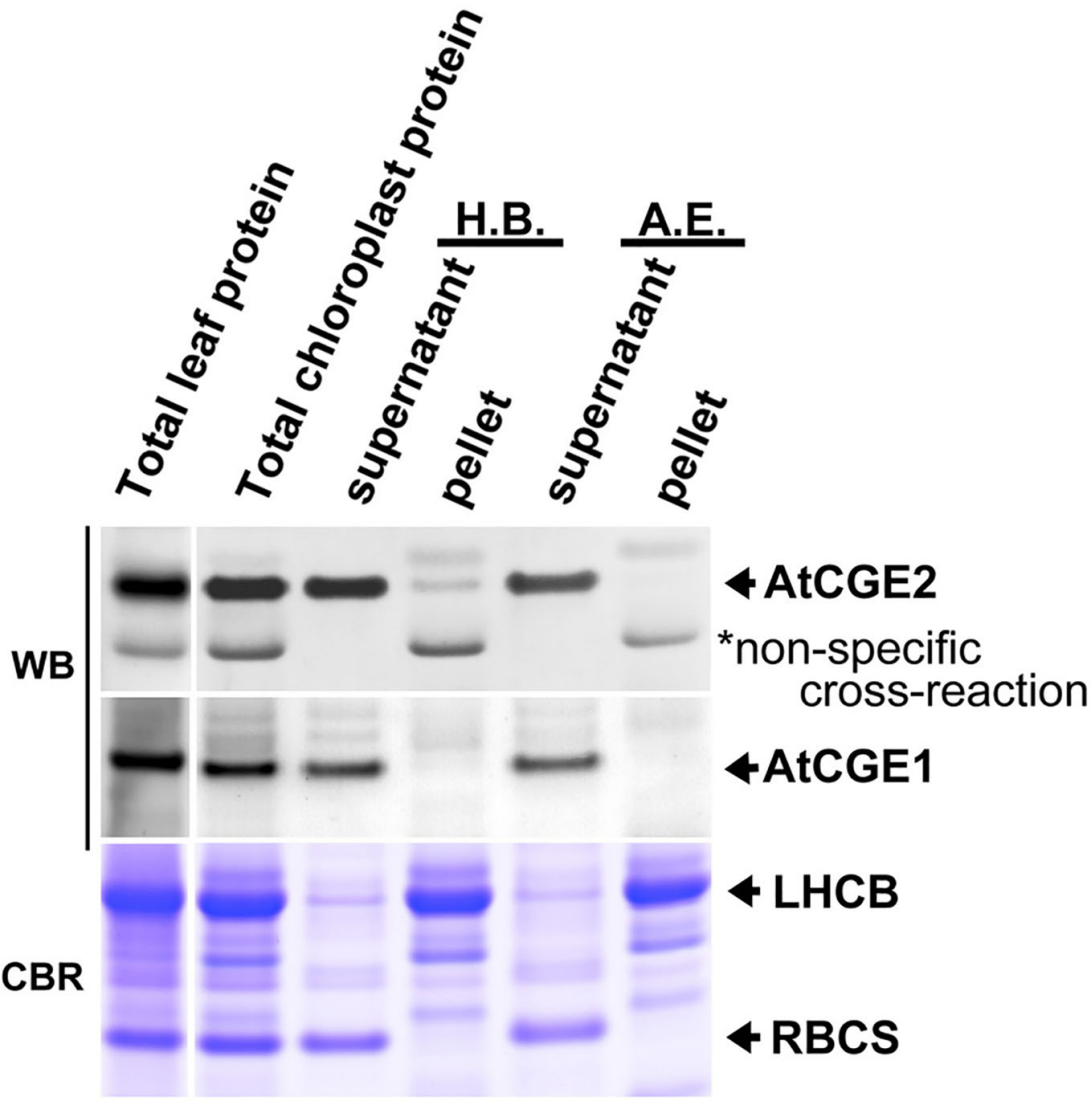
Sub-organellar distribution of AtCGEs. Total chloroplast proteins were fractionated by ultracentrifugation after being lysed with hypotonic buffer (H.B.) or alkaline extraction (A.E.). Samples were resolved by PAGE and visualized by Western blot (WB). Coomassie Blue R-250 (CBR) stained light-harvesting chlorophyll a/b binding (LHCB) proteins and the small subunit of Rubisco (RBCS) were used as membrane and stromal protein control, respectively. Samples were loaded with equal proportion.

We next analyzed the sub-organellar fractionation of AtCGEs. As shown in **Figure 4**, we found that AtCGE1 is exclusively located in the soluble part either fractionated by hypotonic buffer or the alkaline extraction method. In comparison, a portion of AtCGE2 did associate with chloroplast membranes fractionated by hypotonic buffer, but not by alkaline extraction, indicating a possible role of AtCGE2 by peripheral membrane association, such as in protein import (**Figure 3**).

### Functional Complementation of *E. coli grpE* Mutant

**Figure 5:**
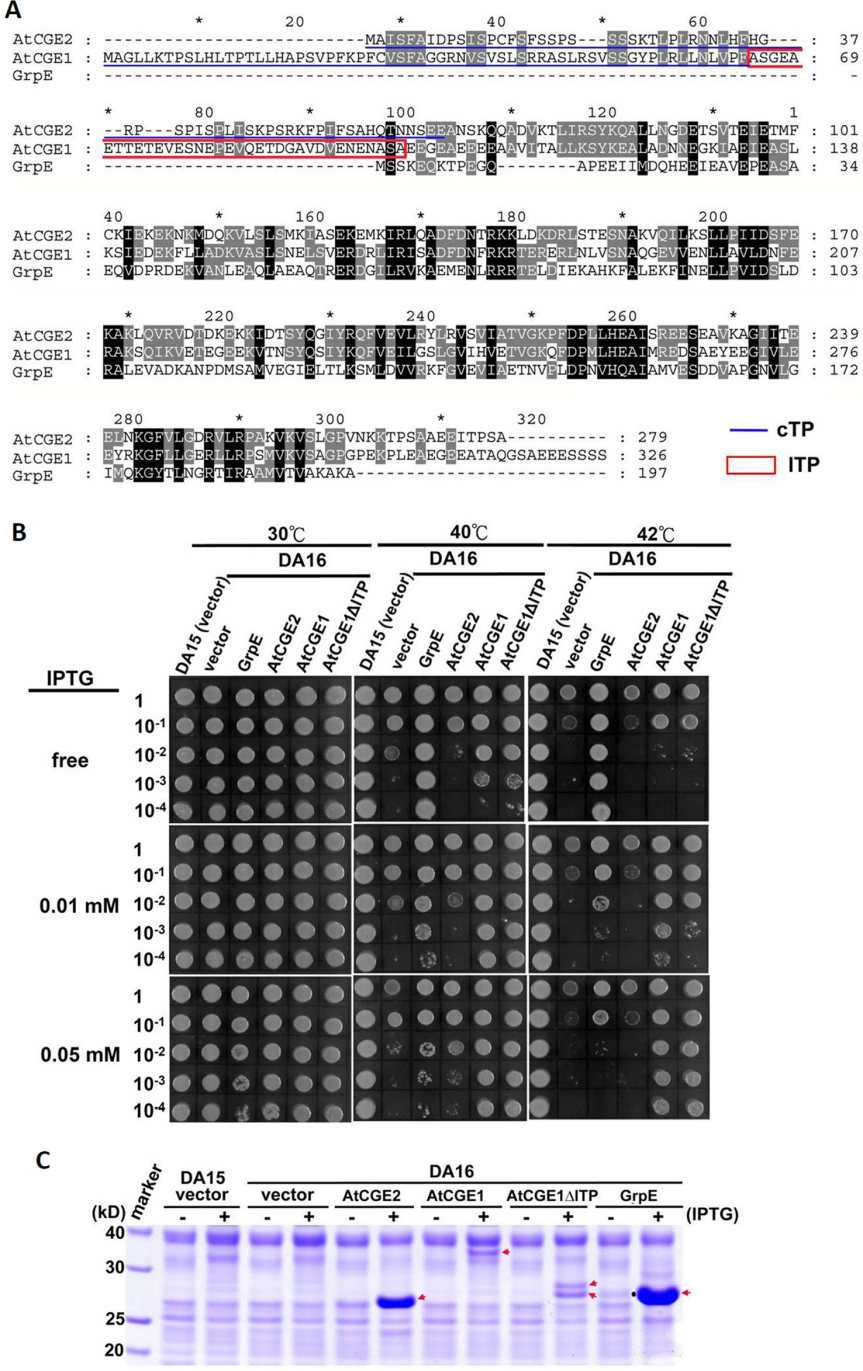
Functional complementation of *E. coli grpE* mutant (DA16) by *AtCGE*s. **(A)** Alignment of GrpE homologues. Underlines represent predicted chloroplast transit peptide (cTP), and a boxed region indicates a putative lumen transit peptide (lTP). **(B)** Complementation of the DA16 mutant by expressing AtCGEs. Overnight liquid culture of DA16 mutant harboring GrpE, AtCGE1, or AtCGE2 was refreshed in LB, and grown to OD_600_ about 0.8. A serial dilution of bacterial suspension (4 μl) was dropped on IPTG-free or -containing LB agar plates for 2 h pre-induction at 30°C, then challenged with different heat shock condition as indicated. DA15 is the wild counterpart of DA16 mutant. **(C)** Induction test of GrpE homologues. Arrows indicates the protein bands induced by IPTG. A dot in GrpE/IPTG-free lane indicates the leaky-expressed GrpE.

*E. coli* DA16 (*grpE280*) is a heat-sensitive mutant carrying a G122D missense mutation in GrpE, which impairs interaction of GrpE with DnaK and the ADP/ATP-exchange activity of GrpE (Ang et al., 1986; Grimshaw et al., 2005). For complementation test, mature AtCGE1 and AtCGE2, in which transit peptides were removed, were expressed in DA16 mutant to determine the degree of thermotolerance. Chloroplast transit peptides (cTP) of AtCGEs were surveyed through the PPDB database (http://ppdb.tc.cornell.edu) (Sun et al., 2009) and predicted by the ChloroP program (Emanuelsson et al., 1999) (**Figure 5A**). Additionally AtCGE1 has a predicted lumen transit peptide (lTP) (the boxed region in **Figure 5A**) annotated in the PPDB database. Therefore, we also constructed an lTP-deleted clone, named AtCGE1ΔlTP. Notably, the current proteomic study indicates that AtCGE1 is a stromal protein (Peltier et al., 2006), and AtCGE1 was not identified in the lumen proteome of Arabidopsis chloroplasts (Schubert et al., 2002). It was found that AtCGE1 (with or without lTP) can efficiently complement the heat-sensitive phenotype of DA16 after IPTG induction. Although AtCGE2 was highly expressed in DA16, no obvious complementation ability could be detected at either 40°C or 42°C (**Figures 5B, C**). It was noted that, for unknown reason, there were two induced protein bands in the AtCGE1ΔlTP construct (**Figure 5C**), and both of them can be recognized by an anti-AtCGE1 antibody (**Figure S3**). It is interesting that *E. coli* GrpE can complement the DA16 mutant even before IPTG induction due to a leaky expression and its complementation ability actually decreased after a higher expression level was induced at a higher IPTG concentration (**Figures 5B, C**). This observation supports the notion that a proper GrpE/DnaK ratio is critical for adequate chaperone activity as described in Sugimoto et al. (2008). Therefore we cannot exclude the possibility that the inability of AtCGE2 to complement the DA16 mutant may be due to its excess expression.

### *In Vitro* Luciferase Refolding Assay

**Figure 6:**
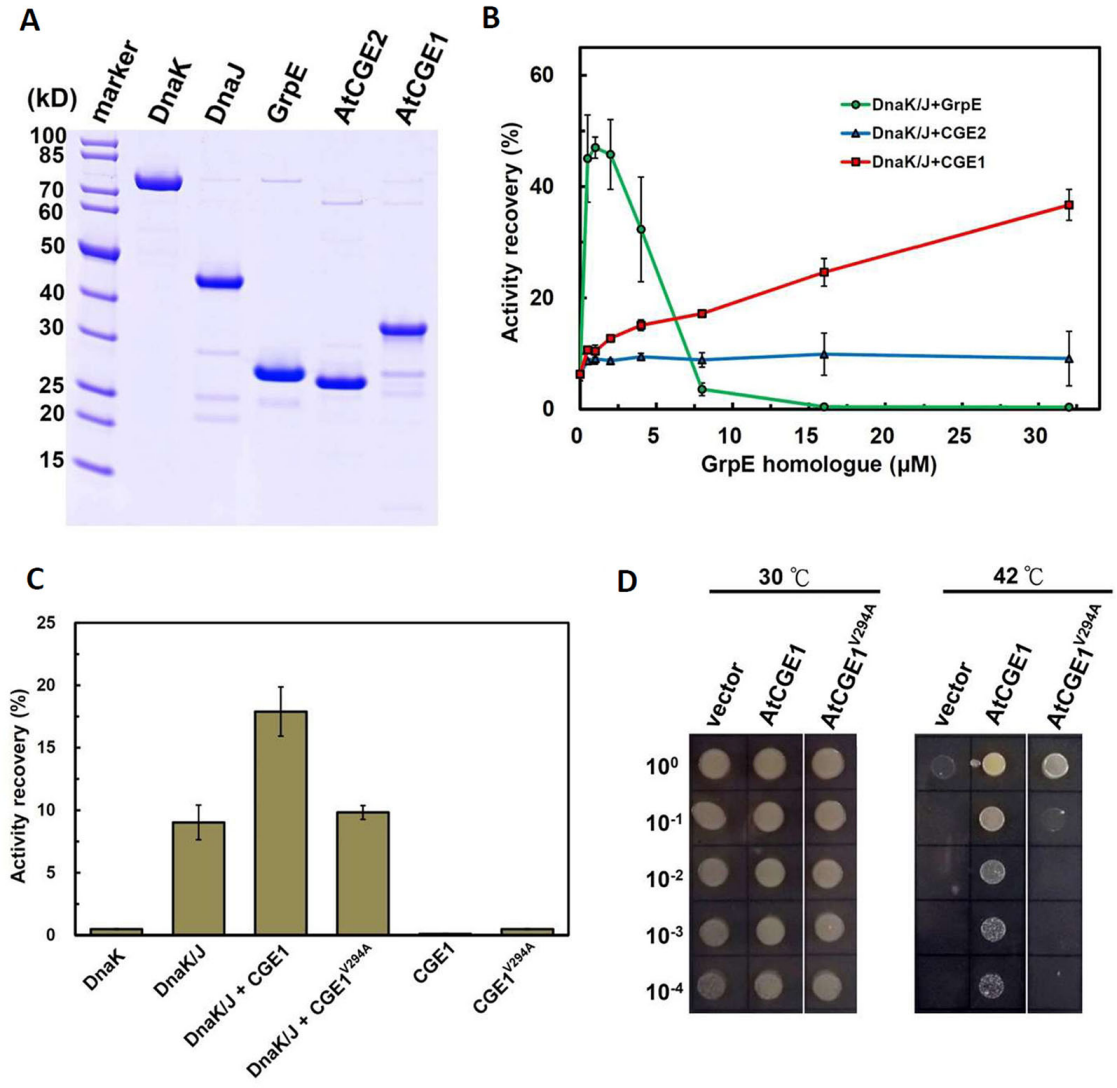
AtCGE1, but not AtCGE2, exhibited GrpE-like cochaperone activity. **(A)** Recombinant proteins of Dnak, DnaJ, GrpE, and AtCGEs were affinity purified, resolved on PAGE, and stained by CBR. **(B)** A dosage response of GrpE homologues on stimulating the luciferase refolding by DnaK/J machinery. DnaK and DnaJ concentrations are 5 and 1 μM, respectively. **(C)** Activity recovery of chemically denatured luciferase by DnaK/J machinery with the stimulation of wild-type or mutated AtCGE1 as indicated. The concentration of DnaK, DnaJ, and AtCGE1 are 5, 1, and 16 μM, respectively. **(D)** *E. coli* DA16 complementation by wild-type and mutated AtCGE1. After 2 h pre-induction on LB plate containing 0.05 mM IPTG at 30°C, the bacteria were challenged at 42°C for overnight.

Functional complementation assay revealed that only AtCGE1, and not AtCGE2, can complement the heat-sensitive DA16 mutant. To further confirm whether the complementation ability is really directly correlated with their co-chaperone activity, their stimulation of luciferase refolding by DnaK/J was analyzed. Recombinant DnaK, DnaJ, GrpE, AtCGE2, and AtCGE1 with His-tag were purified with high purity as shown in **Figure 6A**. Chemically denatured luciferase was refolded by 5 μM Dnak plus 1 μM DnaJ and stimulated by GrpE homologues from 0.5 to 32 μM. Data was represented as percentage luciferase activity recovery. It has been shown that a proper ratio of GrpE to DnaK is critical for chaperone activity of DnaK system (Sugimoto et al., 2008). Results of our GrpE control were consistent with those previously reported. Refolding of luciferase was stimulated at lower GrpE concentration and inhibited by higher GrpE/DnaK ratio (**Figure 6B**). There was no obvious stimulation by AtCGE2 at tested concentrations. AtCGE1 exhibited a concentration-dependent increase in stimulation of luciferase refolding (**Figure 6B**). Even in higher AtCGE1/DnaK ratio, there is no inhibitory effect on DnaK refolding activity, implying that although AtCGE1 has a good co-chaperone activity to DnaK, it may harbor distinct regulatory mechanism to GrpE. Further analyses of reciprocal exchange in chaperone/co-chaperone pairs between DnaK/GrpE and Hsp70/CGE may be needed to clarify this issue in the future. Next, to clarify whether the detected co-chaperone activity of AtCGE1 really originated from our target protein, we tried to make loss-of function mutations on two conserved Val residues of AtCGE1 by site-directed mutagenesis. It is noted that a Val-Lys-Val tri-residue motif is conserved in all plant CGE proteins. Instead, *E. coli* GrpE has a Val-Thr-Val (residues 190–192) tri-residue motif, which is located in the β-domain. Gelinas et al. (2004) have shown that the V192A mutation of GrpE destabilizes the GrpE β-domain and losses its ability to interact with DnaK. The Val294 and Val296 of AtCGE1 are respectively corresponding to the Val190 and Val192 of GrpE. Finally, we only got the V294A mutation of AtCGE1. *In vitro* refolding assay revealed that the co-chaperone activity of AtCGE1^V294A^ was almost totally abolished (**Figure 6C**). *In vivo* complementation test also showed that AtCGE1^V294A^ almost lost its complementation ability (**Figure 6D**). It convinces the important role of the Val294 residue on the co-chaperone activity of AtCGE1 proteins. Taken together, these results support the notion that the detected activity is truly conferred by the co-chaperone activity of AtCGE1, and not caused by contamination of other co-purified proteins with chaperone/co-chaperone activities during purification. Therefore, we suggest that AtCGE1 is a bona fide functional GrpE homologue based on its *in vivo* complementation ability and *in vitro* co-chaperone activity, although it may have evolved different co-chaperone properties from GrpE to cope with the need of chloroplast Hsp70 system. Furthermore, no detectable co-chaperone activity by AtCGE2 implies that its inability to complement the DA16 mutant is most probably due to the lack of co-chaperone activity, and not caused by its excess expression.

### AtCGE1 and AtCGE2 Can Form a Heterocomplex With Co-Chaperone Activity

**Figure 7:**
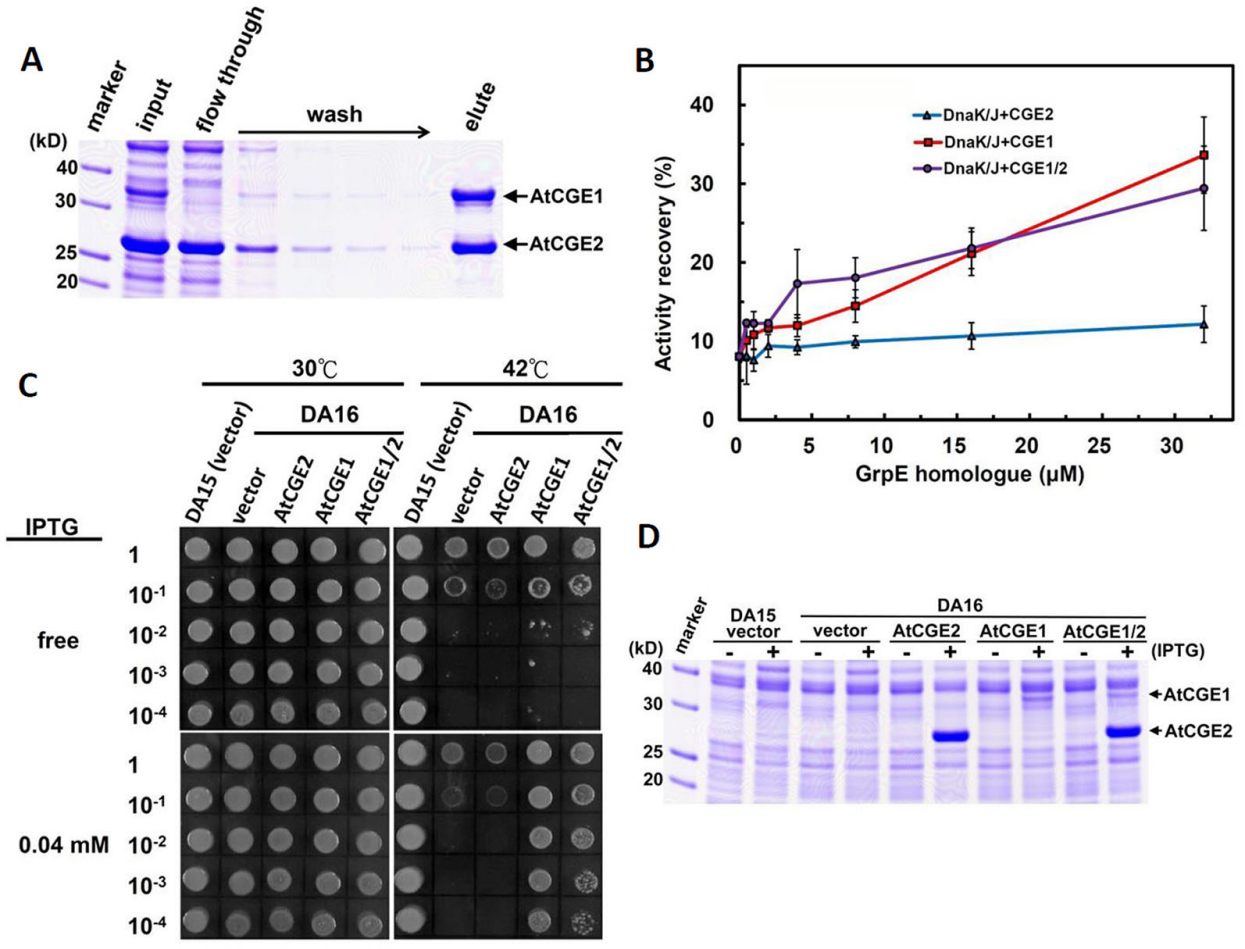
AtCGE1/2 heterocomplex exhibited a comparable cochaperone activity with AtCGE1. **(A)** Affinity purification of AtCGE1/2 heterocomplex. The eluted sample contained AtCGE1 and AtCGE2-6xHis with approximately 1:1 ratio. **(B)** A dosage response of ACGEs on stimulating the luciferase refolding by DnaK/J machinery. DnaK and DnaJ concentrations are 5 and 1 μM, respectively. For AtCGE1/2 heterocomplex, 1 μM protein concentration equals to approximately 0.5 μM each. **(C)** Complementation of *E. coli* DA16 mutant by AtCGEs. **(D)** Induction of AtCGEs by IPTG. Protein bands corresponding to AtCGE1 and AtCGE2 are indicated.

To test whether AtCGE1 and AtCGE2 can form a heterocomplex, we co-expressed AtCGE1 and AtCGE2 in *E. coli* BL21(DE3). We tagged AtCGE1 with 6xHis. As shown in **Figure 7A**, the crude protein extract (see the “input” lane) contained a high AtCGE2/AtCGE1 ratio due to higher expression level of AtCGE2 as observed previously (**Figure 5C**). Following Talon-bead binding, the flow-through sample had no visible AtCGE1 band, implying most AtCGE1 was bound to beads. After stringent wash, AtCGE2 was co-eluted with His-tagged AtCGE1 with a molar ratio approximately 1:1, indicating a robust formation of heterocomplex between AtCGE1 and AtCGE2. *In vitro* luciferase refolding assays demonstrated that AtCGE1/2 heterocomplex efficiently stimulated DnaK/J-mediated luciferase refolding with comparable activity to that of AtCGE1 homocomplex (**Figure 7B**). Further, *in vivo* complementation assays also showed that co-expressed AtCGE1/2 functioned similarly to AtCGE1 in rescuing the DA16 heat-sensitive phenotype (**Figures 7C, D**).

### AtCGE1 and AtCGE2 Undergo Homo- and Hetero-Oligomerization

**Figure 8:**
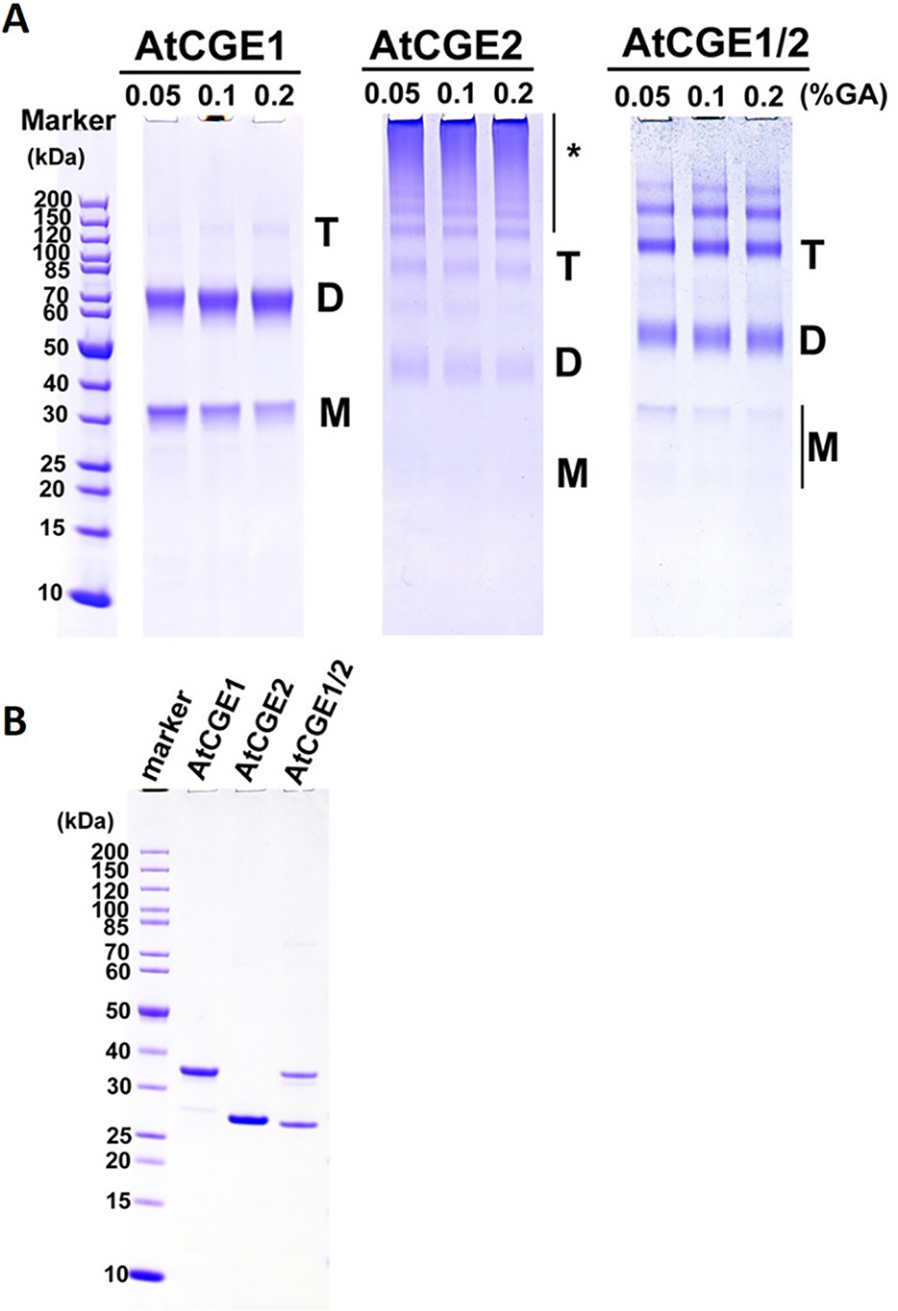
Oligomeric states of AtCGEs. **(A)** 5 μM proteins in common buffer were crosslinked with glutaraldehyde (GA) at 0.05, 0.1, and 0.2% for 10 min. After crosslinking, their oligomeric states were visualized by 4–12% SDS-PAGE analysis and CBR staining. **(B)** Purified AtCGE proteins were mock incubation in common buffer, and were resolved on SDS-PAGE as uncrosslinked controls. For each lane, 1 μg protein was loaded. Asterisk (*) indicates high molecular mass oligomers.

In bacteria, GrpE is known to form a functional homodimer and to further oligomerize into tetramer, hexamer, and high molecular mass oligomers upon the increase of concentration (Wu et al., 1996; Harrison et al., 1997). It has been shown that Arabidopsis CGE1 and CGE2 can form homo- and hetero-dimers *in vivo* by BiFC experiments (de Luna-Valdez et al., 2019). To determine the oligomeric state of Arabidopsis CGE proteins, 5 μM each of purified AtCGE1, AtCGE2, and AtCGE1/2 were crosslinked by glutaraldehyde at 0.05 to 0.2% for 10 min. As shown in **Figure 8**, AtCGE2 was prone to aggregate to form unusually high molecular mass oligomers, with only a few dimers and tetramers. Monomers were nearly undetectable. This tendency to aggregation may be one of the reasons that AtCGE2 was inactive in co-chaperone activity assays (**Figures 6B, C**). AtCGE1 was detected largely in a dimeric homocomplex, with a small proportion in the monomeric form. Interestingly, when AtCGE1 and 2 were co-expressed, they formed dimers and tetramers. The size of the dimers was in between the AtCGE1 homodimer and the AtCGE2 homodimer (**Figure 8**), suggesting that they were AtCGE1/2 heterodimers. The size of the tetramers was also in between the AtCGE1 homotetramer and the AtCGE2 homotetramer, and the aggregation of AtCGE2 at the high molecular mass region was no longer observed. This result was in agreement with the result of AtCGE2 binding to AtCGE1 at a molar ratio of approximately 1:1 (**Figure 7A**) and both results suggest that AtCGE1 and AtCGE2 have a high propensity to form heterocomplexes. To sum up, both *in vivo* BiFC (de Luna-Valdez et al., 2019) and our *in vitro* crosslinking analyses support that AtCGE1 and AtCGE2 can form homo- and hetero-oligomers, although further fractionation of different homo- and hetero-oligomers to correlate their biological activities and in combination with a more precise way to determine their oligomeric status (such as analytical ultracentrifuge) may be needed to clarify every detail.

## Discussion

Gene and genome duplications are the major driving forces of gene diversification and evolution (Lynch and Conery, 2000). de Luna-Valdez et al. (2019) recently reported that the CGE protein sequences from embryophytes form a monophyletic group that further divides into two subgroups, naming type A and type B. The two subgroups of CGEs can be distinguished from each other by variations in short motifs conserved in CGE proteins. Our phylogenetic analysis showed that flowering plants did evolved two distinct clades of CGE homologues prior to the divergence of monocot and dicot lineages, and functional characterizations suggest that two Arabidopsis CGE proteins have gained diversified activities during evolution. Although the early diverging land plant, *Physcomitrella patens*, also has two copies of CGE proteins, these two CGEs are highly similar with 85% sequence identity in their mature protein regions, and are grouped into the same clade. Shi and Theg (Shi and Theg, 2010) reported that single *Physcomitrella cge1* and *cge2* mutants are viable on phototrophic media but displayed similar slow growth phenotypes and are delayed in development of leafy shoots compared with the wild type. Their further characterizations revealed that *cge1 cge2* double-knockout mutant is lethal, indicating the functional redundancy between these two CGE proteins in moss. These two moss CGE proteins might have originated from a large-scale gene duplication event, possibly involving the whole genome, occurring in *Physcomitrella patents* (Rensing et al., 2008). But green algae and the lycophyte *Selaginella moellendorffii* generally have fewer genes per shared gene family when compared to angiosperm species. It is interesting that most flowering plants have also evolved at least two mitochondrial GrpE homologues (MGEs), for which duplication occurred independently after the divergence of many distantly related plant species. In Arabidopsis, it has been demonstrated that the two MGE homologues (AtMGE1 and AtMGE2) are derived from a recent gene duplication and AtMGE2 is sub-functionalized to confer thermotolerance to chronic heat shock (Hu et al., 2012). In comparison to the nature of the single GrpE homologue in bacteria and yeast mitochondria, we suggest that there is a convergent trend in gene duplication and functional/activity diversification both in mitochondria and chloroplasts of flowering plants, which should be beneficial to accommodate the changing environment on the land; although two CGE clades are derived from an ancient duplication, two MGE homologues were duplicated more recently.

GrpE homologues are nucleotide exchange factors, which stimulate the exchange of Hsp70/DnaK-bound ADP with ATP to complete the ATP-dependent binding and release of polypeptides (Young, 2010). Unlike DnaJ homologues, which can broaden the functions of the Hsp70 machinery by presenting the machinery with different client proteins (Kampinga and Craig, 2010), there is no substrate specificity mediated by GrpE homologues. It is unclear how a duplicated GrpE homologue confers diversified biological functions and co-chaperone activities in the organelles of flowering plants. One of the likely means is through different spatial or temporal expression patterns of the duplicated homologue. This might happen in plant mitochondrial *MGE*s, which are differentially induced by heat shock and UV-B irradiation (Hu et al., 2012). However, two *CGEs* are expressed in a similar pattern without large differences during development or upon abiotic stress, except in dry seeds and in recovering heated roots (see microarray database by Arabidopsis eFP browser at http://bar.utoronto.ca/efp_arabidopsis/cgi-bin/efpWeb.cgi). Our biochemical analyses suggested that the two distinct clades of plant CGEs may modulate Hsp70 machinery by diversified co-chaperone activity and membrane association. According to *grpE* complementation and luciferase refolding assays, we showed that only AtCGE1, but not AtCGE2, can complement the heat-sensitive phenotype of a *grpE* mutant, and significantly stimulate the luciferase refolding mediated by the DnaK/J machinery. These results indicate that AtCGE1 functions as the principle GrpE homologue. Although AtCGE2 homocomplex shows no obvious co-chaperone activity, its hetero-oligomers complexed with AtCGE1 exhibit a comparable co-chaperone activity to the AtCGE1 homocomplex. For this reason *AtCGE2* is dispensable under normal growth conditions, but becomes more important when *AtCGE1* is knocked down. Furthermore the unique properties of AtCGE2 may offer a way to fine-tune the balance of ATP/ADP-bound state of Hsp70. Indeed, it has been shown that a proper ratio of GrpE to DnaK is important for the chaperone activity both *in vitro* and *in vivo* (Sugimoto et al., 2008). Overexpression of DnaK or GrpE alone will disturb the balance between the ATP-bound (low affinity to substrate) and the ADP-bound state (high affinity to substrate) of DnaK, leading to inefficient chaperone activity (Blum et al., 1992; Sugimoto et al., 2008). To avoid the negative effect caused by a high dosage of GrpE homologues, flowering plants may have evolved a fine-tuned mediator of CGE homologues. It is possible that AtCGE1 homocomplex takes charge of the principle role in stimulating the ATP cycles of chloroplast Hsp70, and inactive AtCGE2 may become active through hetero-oligomerization with AtCGE1 in a certain conditions.

Recently it has been shown that chloroplast Hsp70 is important for protein import into chloroplasts (Shi and Theg, 2010; Su and Li, 2010), and *Physcomitrella* CGE proteins are involved in the process possibly by mediating the release of precursor proteins from Hsp70 (Shi and Theg, 2010). Our *in vitro* import assays showed that AtCGE1 is required for protein import into chloroplasts, and AtCGE2 could have a similar function at a quite lower level. Furthermore, our sub-organellar fractionations found that AtCGE2, but not AtCGE1, can associate with chloroplast membranes to some degree. Although the mechanism for membrane association of AtCGE2 is still not known, it can be imagined that AtCGE2 may have a more important role in chloroplast membrane biology. Considering the nature of protein import, in which Hsp70 may hold the newly imported precursors near the inner envelope membrane for a longer time to avoid their sliding back to the cytosol, membrane associated AtCGE2 with a low ADP/ATP exchange activity may be beneficial for keeping chloroplast Hsp70 in a high-affinity ADP-bound state. Once the precursor proteins reach the stroma, AtCGE1 may efficiently promote their release from Hsp70s by its highly-active co-chaperone activity. However, the detailed mechanism will require further investigation, especially in providing a direct *in vivo* evidence for Hsp70/CGE interaction.

Our mutant studies showed that embryo development of atcge*1* was arrested at an early globular stage, indicating the essential role of AtCGE1. Our result is consistent with the data in the SeedGenes database (http://www.seedgenes.org) based on the study of essential genes in Arabidopsis conducted by Meinke et al. (2008), in which AtCGE1 mutant was designated as *emb1241*. This again emphasizes the pivotal function of chloroplast Hsp70 machinery. Our previous report showed that chloroplast Hsp70 is essential for plant development, and *cphsc70-1 cphsc70-2* double mutation is lethal to embryos (Su and Li, 2008). It is of interest why chloroplast Hsp70 machinery is vital at such an early stage of development. Recently, non-randomly distributed chloroplast-containing cells were found as early as the globular stage of embryogenesis in Arabidopsis by the observation of chlorophyll fluorescence (Tejos et al., 2010), and well-developed chloroplasts with grana stacks were seen in the peripheral endosperm of developing seeds (Belmonte et al., 2013). Through data mining of transcriptomic profiles of Arabidopsis developing seeds generated by Belmonte et al. (2013), we found that *AtCGE1* was highly expressed from the preglobular to the heart stage, particularly in the peripheral endosperm of developing embryos (Figure S4). In addition, the embryogenesis of the chloroplast protein import mutants, *tic110* and *toc75-III*, cannot proceed beyond the globular stage (Baldwin et al., 2005; Kovacheva et al., 2005), suggesting that the cessation of embryo development caused by *atcge1* mutation may also mainly be due to the defect of protein import, leading to hindrance of plastid/chloroplast biogenesis. A further proteomic study on identify the client proteins chaperoned by Hsp70/CGE machinery will be helpful to screen the putative molecules related to the embryo lethality of *atcge1* mutant.

## Data Availability Statement

All datasets generated for this study are included in the article/**Supplementary Material**.

## Author Contributions

P-HS and H-YL conducted the biochemical analyses. P-HS and Y-HL contributed to the isolation and characterization of Arabidopsis mutants. P-HS designed the experiments and wrote the manuscript.

## Funding

This work was supported by the funding of Ministry of Science and Technology (Grant NSC100-2311-B-001-029) and Academia Sinica to P-HS.

## Conflict of Interest

The authors declare that the research was conducted in the absence of any commercial or financial relationships that could be construed as a potential conflict of interest.
