# Supplementary material for: Two Arabidopsis Chloroplast GrpE Homologues Exhibit Distinct Biological Activities and Can Form Homo- and Hetero-Oligomers
Source: Front Plant Sci. 2020 Jan 22;10:1719. doi: 10.3389/fpls.2019.01719 (PMC6987454; doi:10.3389/fpls.2019.01719)
Supplement: Supplementary file 1 [file DataSheet_1.pdf]

Supplementary Material

Table S1. Genomic resources used in this study.

Organism	Abbreviation	Source[*]
Arabidopsis lyrata	Al	NCBI[a]
Arabidopsis thaliana	At	NCBI
Aegilops tauschii	-[**]	NCBI
Amborella trichopoda	Atr	NCBI
Brachypodium distachyon	Bd	Ensembl_Plants[b]
Brassica rapa	Br	Phytozome[c] (JGI)
Cicer arietinum	Ca	NCBI
Citrus clementina	-	NCBI
Cucumis melo	Cm	NCBI
Chlamydomonas reinhardtii	Cr	NCBI
Capsella rubella	Cru	NCBI
Cucumis sativus	Cs	NCBI
Citrus sinensis	-	NCBI
Chlorella variabilis	Cv	JGI[d]
Eucalyptus grandis	-	NCBI
Erythranthe guttata	-	NCBI
Eutrema salsugineum	Es	NCBI
Fragaria vesca	Fv	NCBI
Glycine max	Gm	NCBI
Hordeum vulgare	Hv	NCBI
Jatropha curcas	Jc	NCBI
Micromonas pusilla CCMP1545	Mp	JGI[d]
Medicago truncatula	Mt	NCBI
Oryza brachyantha	Ob	Ensembl_Plants
Oryza sativa Indica Group	-	NCBI
Oryza sativa Japonica Group	OsJ	Rice Genome Annotation Project[e]
Ostreococcus lucimarinus	Ol	JGI
Ostreococcus tauri	Ot	NCBI
Physcomitrella patens	Pp	NCBI
Prunus persica	Ppe	NCBI
Populus trichocarpa	Pt	NCBI
Phaseolus vulgaris	Pv	NCBI
Ricinus communis	-	NCBI
Sorghum bicolor	Sb	NCBI
Setaria italica	Si	Ensembl_Plants
Selaginella moellendorffii	Sm	Phytozome (JGI)
Solanum tuberosum	St	NCBI
Synechocystis	Syn	NCBI
Triticum aestivum	Ta	Ensembl_Plants / NCBI
Theobroma cacao	-	Phytozome (JGI)
Vitis vinifera	-	NCBI
Volvox carteri	Vc	Phytozome (JGI)
Zea mays	Zm	Phytozome (JGI)

[*]After surveying species genome databases, those CGE sequences with corresponding accession numbers in NCBI database were used with priority.

[**]Abbreviation not used in this study.

[a]National Center for Biotechnology Information, http://www.ncbi.nlm.nih.gov/

[b]http://plants.ensembl.org/

[c]The Plant Genomics Resource, http://phytozome.jgi.doe.gov/pz/portal.html

[d]The DOE Joint Genome Institute, http://genome.jgi.doe.gov/

[e]http://rice.plantbiology.msu.edu/

Table S2. Oligonucleotide primers used in this study.

Name	sequence (5' to 3')
CGE1-1130-AS	TCTCTGTATCTCTCTCACAC
CGE1-998-S	AAAGCCCCTTGAAGCTGAAG
CGE1-AS	CCTCTCCCTCTTTTATCTCTG
CGE1E1-S	GCTTACTAAACCTCGTAC
CGE1-I2-AS	CACCAGCAAGCTGAGCAAC
CGE1-NdeI-S	ACCTCGTACATATGGCTTCGGGAGAAGCTGAG
CGE1P-S	CGTTGCACCCACTACTTCTG
CGE1-R287A-AS	GGACGTAAAAGCGCTTCACCTAGCAAG
CGE1-R287A-S	CTTGCTAGGTGAAGCGCTTTTACGTCC
CGE1t-AS	GTACCCAAAGAACCCTAACC
CGE1-V294A-AS	CAGCTGATACTTTCGCCATTGAAGGAC
CGE1-V294A-S	GTCCTTCAATGGCGAAAGTATCAGCTG
CGE1-XhoI-AS	CTTCACTCGAGAGATGAAGATGATTCTTCTT
CGE2P-S	CTCTTTCTCCTTACCAATCAC
CGE2-AS	CTCTGAAGCCAAAGAACTTTC
CGE2-NdeI-S	TAACAGTCATATGGCGAATTCAAAGCAGCAAG
CGE2-S	GGGACTCACTGAGATTACTAC
CGE2t-AS	TGTTGTTGTGTGAGAGATGC
CGE2-XhoI-AS	ACATCGCTCGAGAGCAGAAGGTGTTATTTCC
LBa1-2	GGTGATGGTTCACGTAGTG
pDS-LoxHs-L4'	ATGTAGATTTTCCGGACATG
Tag5	CTACAAATTGGTTTTCTTATCGAC
XhoI-CGE1-AS	GGTACCCTCGAGAGATGAAGATGATTCTTCTTC
XhoI-CGE1-S	GGAAACCTCGAGTAAGAGCTCAATAATTTTGTTTAAC

Figure S1

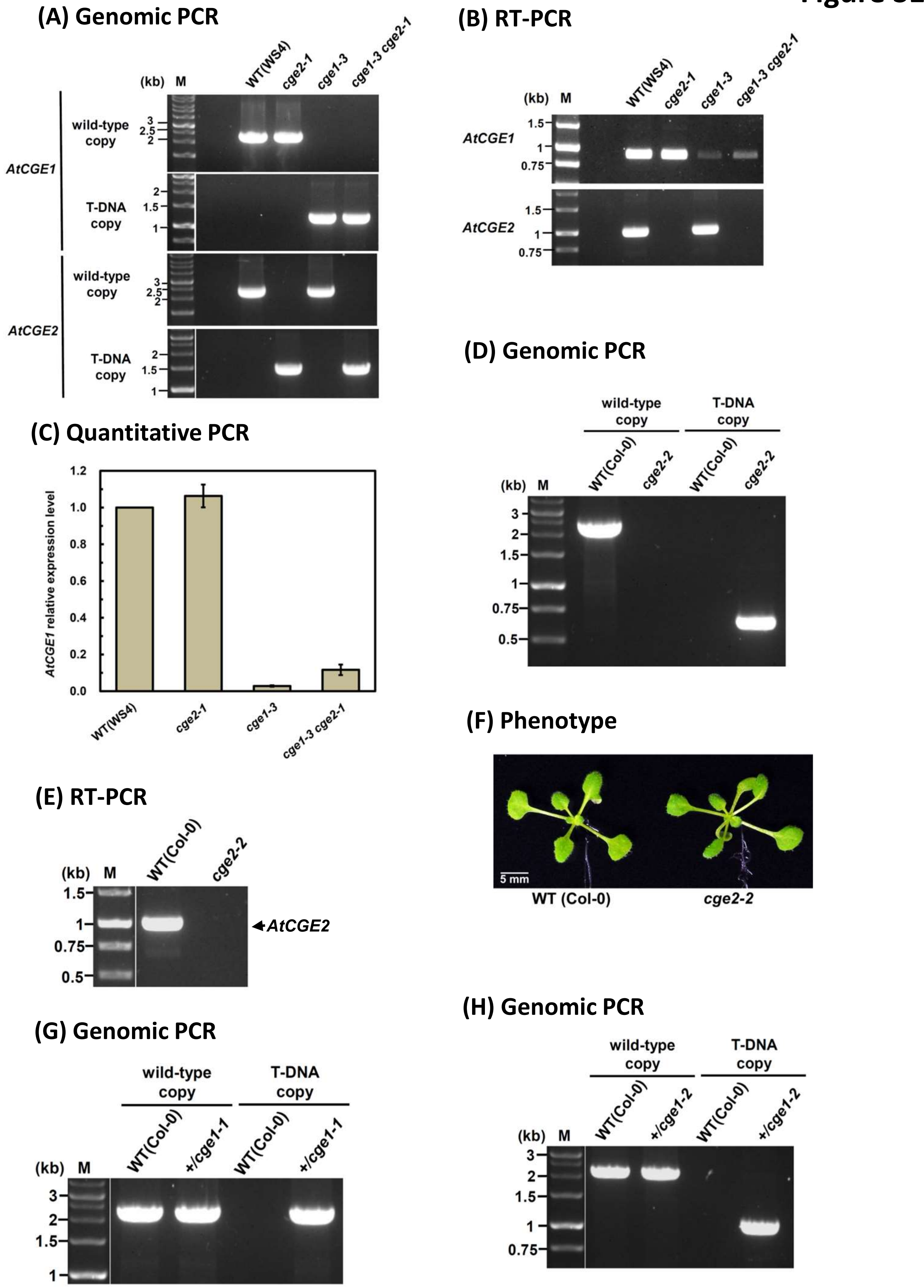
(A) Genomic PCR
(kb) M
WT(WS4)
cge2-1
cge1-3
cge1-3 cge2-1
AtCGE1
wild-type copy
3
2.5
2
T-DNA copy
2
1.5
1
AtCGE2
wild-type copy
3
2.5
2
T-DNA copy
2
1.5
1
(B) RT-PCR
(kb) M
WT(WS4)
cge2-1
cge1-3
cge1-3 cge2-1
AtCGE1
1.5
1
0.75
AtCGE2
1.5
1
0.75
(C) Quantitative PCR
AtCGE1 relative expression level
1.2
1.0
0.8
0.6
0.4
0.2
0.0
WT(WS4)
cge2-1
cge1-3
cge1-3 cge2-1
(D) Genomic PCR
wild-type copy
T-DNA copy
(kb) M
WT(Col-0)
cge2-2
WT(Col-0)
cge2-2
3
2
1.5
1
0.75
0.5
(E) RT-PCR
(kb) M
WT(Col-0)
cge2-2
1.5
1
0.75
0.5
AtCGE2
(F) Phenotype
5 mm
WT (Col-0)
cge2-2
(G) Genomic PCR
wild-type copy
T-DNA copy
(kb) M
WT(Col-0)
+/cge1-1
WT(Col-0)
+/cge1-1
3
2
1.5
1
(H) Genomic PCR
wild-type copy
T-DNA copy
(kb) M
WT(Col-0)
+/cge1-2
WT(Col-0)
+/cge1-2
3
2
1.5
1
0.75

Figure S1. Identification of *atcge* mutants. (A, B) Confirmation of *atcge1-3*, *atcge2-1* and *atcge1-3 atcge2-1* mutants by genomic PCR and RT-PCR as indicated in WS background. (C) Relative expression levels of *AtCGE1* in mutants as indicated by quantitative PCR with primers of CGE1-998-S and CGE1-1130-AS. (D, E) Genomic PCR and RT-PCR validation of *atcge2-2* mutant in Col-0 background. (F) Phenotype of *atcge2-2* grown on agar-solidified MS medium containing 2% sucrose for 14 days. (G, H) Confirmation of *atcge1-1* and *atcge1-2* heterozygous mutants by genomic PCR.

Figure S2

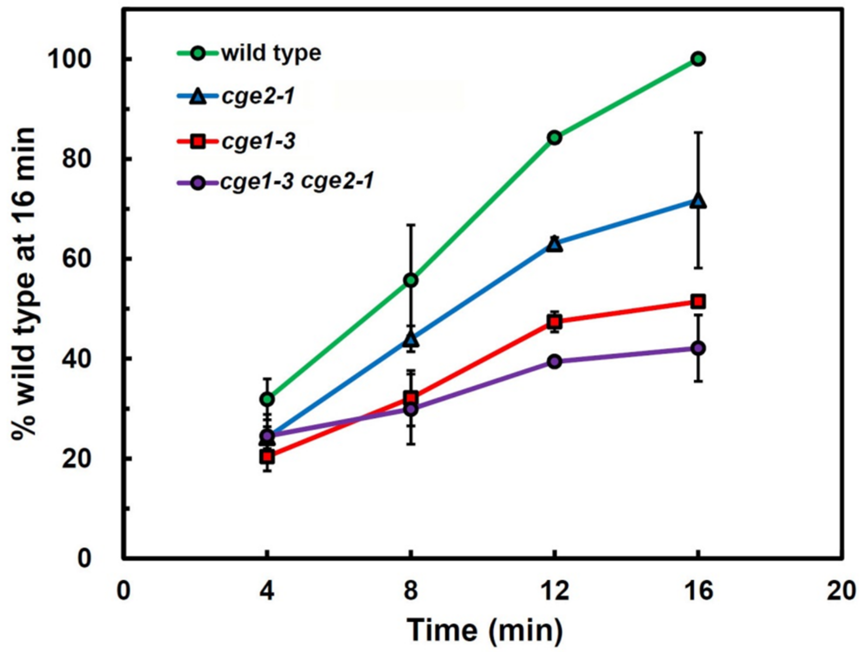

Figure S2. The quantitative result of chloroplast protein imports as shown in Figure 3. Imported mature [^{35}S]Methione-RBCS was quantified using a phosphor imager and normalized to the amount of endogenous light-harvesting chlorophyll a/b binding (LHCB) proteins obtained by scanning the Coomassie-stained gels. The amount of mature RBCS imported in the wild type at 16 min was set as 100%. Data shown in the graphs are means ± SD of two independent experiments.

Figure S3

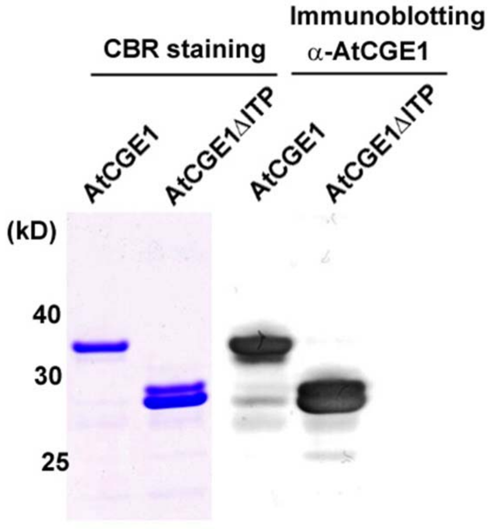

Figure S3. Immunoblotting of purified AtCGE1 and AtCGE1ΔlTP proteins. AtCGE proteins were purified by Talon beads from *E. coli* DA16 expressing the AtCGE1 and AtCGE1ΔlTP constructs. After resolving by PAGE and transblotting onto PVDF membranes, protein bands were visualized by CBR staining or immunoblotting with anti-AtCGE1 antibody.

Figure S4

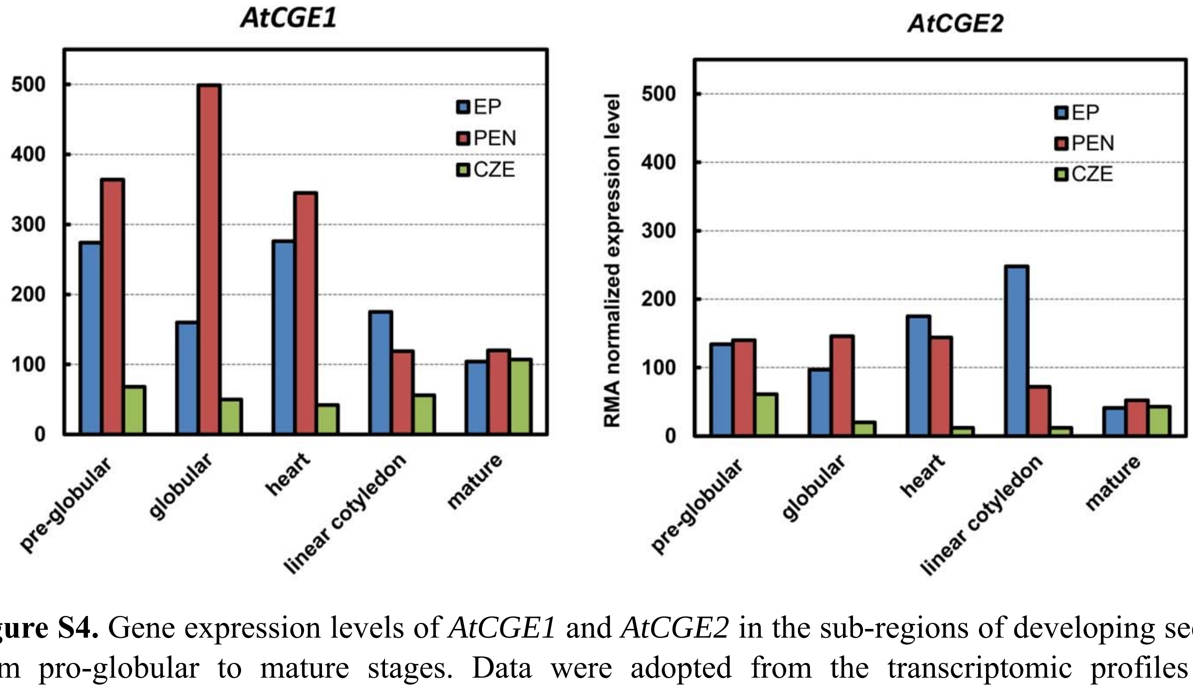

Figure S4. Gene expression levels of *AtCGE1* and *AtCGE2* in the sub-regions of developing seeds from pro-globular to mature stages. Data were adopted from the transcriptomic profiles of Arabidopsis developing seeds generated by Dr. Harada lab. (37). Expression levels are normalized by the algorithm of RMA (Robust Multi-array Average).

Data S1: Sequences of species CGE proteins aligned by ClustalW in fasta format used for phylogenetic analysis. The symbol “-“ represents a gap in multiple alignment.

>SynGrpE(BAA10291)
---MNEDQVSLENQTNPVSPEASDVPAVTPEESPQPTDAVLGEPSGEQSEDPRI
GAATETEGGPLEQEKSSEEIIAILQKDLASHRQELAEQSEQLDSIKKRYVALAAEFDNFRKRTQREKEEQAKLIK
GRTITELLPVVDNFERARTQIKPNSDGENQIHKSYQGVYKNLVDSLKGLGVAPMRPEGKPFDPKYHEAMLREP
TAEYPEDTVIEELVRGYLLDDI-VLRHSMVKVAVAPEEGAEV-----------VNGEAGANP---------------------
>CrCGE1a(AF406935_1)
---AAEEAEAAAAPAEEAAATPLERAKKALDSETLDKDVLT
AALAELEAEMGRLQSAANEANDRAKSLEASLASAKDQYLRLNADFDNFRRRTREESAALTDSVRGDVIKEML
PIVDNFELARTQVKAETEAEQKINNSYQGLYKQMVDLMRTQGVEAVPTTGTPFDPNIHDAIMREPSNSHPDGT
VLQEFRKGFAIGGK-LIRPAMVKVSYTEDGPAAS-----------SEE----------------------------
>CrCGE1b(AF406936_1)
---AAEVQEAEAAAAPAEEAAATPLERAKKALDSETLDKDV
LTAALAELEAEMGRLQSAANEANDRAKSLEASLASAKDQYLRLNADFDNFRRRTREESAALTDSVRGDVIKE
MLPIVDNFELARTQVKAETEAEQKINNSYQGLYKQMVDLMRTQGVEAVPTTGTPFDPNIHDAIMREPSNSHPD
GTVLQEFRKGFAIGGK-LIRPAMVKVSYTEDGPAAS-----------SEE----------------------------
>VcCGE1(PID_109146)
---AAEVQDAPAEAADAPTLSPLDRAKKALEDGSLDKEVL
SGCLAELELEMERMRAEASATMERASALEASANSAKDQYVRLTADFENFRRRTREENAQLTDNVRGDVIKEL
LPIVDNFELARTQVKAETEGEAKINNSYQGLYKQMVDMMRSLGVEAVPTTGTAFDPNIHDAIMREPSNSHPDG
TVLQEFRKGFSIGGK-LLRPAMVKVSYTEEGPANS-----------SEE----------------------------
>CvCGE1(PID_JGI135822)
---AAEAVVPMADSLIERAKQVVETNFVDEAFSETFV
EELEAELAALRTRAEQAAAENTKLEETLLDTRDKFLRLQADFDNFRKRTAGEKDALRVSVRGDTVAELLPLVD
NFELAKAQLKLETEGEKRVDAAYQGLYKQMVELFRGLGLEAVPGVGSPFDPNLHDAIMREASEDVPDGTVLE
EFRKGFVIGDK-LLRPAMVKVSYSDAPAAAA-----------AASSEEAAAGGEVAAPSDSE-----------
>OtCGE1(XP_003080808)
---SSEEEGETAEAVDANDEDEDIVDVEETIESEAHGEESELS------KLLGQLDV
VVG------DNAEAREILALLKTEMGDANAKMVGMEDQVGAMKDQYLRLNADFDNFRKRTAKEKADAANTAK
GAFVKAMLPVLDNFDLAEKNIKGNNEGEEKILTGYQNIVKQMYEIFESQGLVTVPGVGEKFDPMDHEAIMREE
TDEVEEETIIEEFRKGYKIGDS-LIRPSMVKVSTKP---
>OlCGE1_(Chr8_PID_33103)
---AGEEEGETVETEEEVPEVEVVEDDEEVAGGAEEEETALS------ALVGRLEV
VVG------DNADAAELVASLKGEIGDANAKMVGMEDQVAAMKDQYLRLNADFDNFRKRTLKEKENLASSAKG
DFVKALLPVLDNFDLAEKNIKGSTEGEEKILTGYQNMHKQLMEILSSQGLQVVAGVGEPFDPNDHEAIMREEN
DEMDEDTIIEEFRKGYKIGSS-LIRASMVKVSTKP--
>MpCGE_CCMP1545(scaffold_5_PID_33581)
--AAGGEGEEDDEENQRREAEAEVEVVDDDEDEDEDDEDEGSAPPPPSKCEEL
IAKLEESMDA----PELAELAAEVKAIETAFKDLQAANVGLEDQTGALKDQYLRLNADFDNFKKRTIKEKEQLAT
NAKSRVFEAMLPALDNFDLAKANLKTENEGEEKIAKSYEGLVDGLMTILSAQGLSTVAGVGSPFDPNFHEAIM
REESEEHPEDTISEEFRKGYKMGEDQLVRAAMVKVSSGPPASEE---
>PpCGE1(ADB23407)
---------------------------------SSHYRRPVFVAQNSAQTEETEVGEDVETNEEALAEEESASNDASQSPAAEEQSSSIK
SLLEAYREAVAAD--DEGAISDVESQLEAIANERDSLGLKVNSLIEEISTNKDRYLRLNADFDNYRKRSERDRLAT
AGNVRGEVIESLLPMVDNFERAKTSIKTETEAEQKIDXAYQGIYKQFVEIMKSLGVVAVETVGKPFDPNLHEAI
MREDSTEFAEDVVSQEFRRGFRIGDR-LLRPAMVKVSSGPGPAAATDT---------DLPIEEALANE--------------------
>PpCGE2(ADB23408)
--AEKKGEAQVDNVSEASAAEETSSSIKSLLEAYREAVAVN--D
EEAITDVESQLEAIAIERDSLAENANALIGEVSTNKDRYIRLNADFDNYRKRSERDRLATAGNIRGEVVESLLPI
VDNFERAKTSIKTETEGEQKIDNAYQSIYKQFVEIMKSLGVVAIETVGKSFDPNLHEAIMREDSTEFAEDIVSQE
FRRGFRIEDR-LLRPAMVKVSSGPG--PAADT---------DLPIEESLANE--------------------
>SmCGE1(13256)
---AEQVEDEKEKADDDGLEDTTSQVNGSEPTEVSASVIEVLLRNYSEA
VLGN--DQAAMSTIQAELEVIQKERDSLSQLVANLTEESALAKERLLRLNADFDNFRKRSGREKDSLRETVKGD
VVESLLPMIDNFERAKGAIKAETDGERKIDSSYQGIYKQFVDIMKSLGVKVIDTVGKEFNPELHEAIMREESSE

YDEGIVTQEFRRGFLLGEK-LLRAAMVKVSSGKQSNSPA-----------AAPQDSEETTPSDETPVDENSA---------
>AtrCGE2(XP_011624342)
--AAVKVADTHSKRIEGEEDGEISEEESENEAVNEEPQPVITLFHAYKEAI
VEG--DLHKISDIETKLYAIETEKNALEQQITSLSAELSSGKEKFLWLKADFENFRKRSEKDRLTLTSDVQGDVIES
LLPMVDNFERAKQQLKPETEKEKKIDGSYQGIYKQFVEIMRSLKVAVVETVGKPFDPSLHEAIAREESVEYKEG
IIIQEFRRGFVLGDR-LLRPAMVKVSAGPGPRKPS-----------PGSPKPKPTEQLGGVQDSSVSPQPSEVGNI-
>TaCGE2A(Traes_2AL_18913037F)
--VAGADPQVVNGEDFPPMNDLIRLYKKAFLDG--NED
VVSDIEKAITSMEEERSKSASQFESITAEIASGKNKFLRLNADLENFRKQTEKDRAKFTSNIQVELVQSLLPLVDS
FEKTNVEITLETEKEQKISASYQGIYKQLVETLKNLGVGVVETVGKPFDPVVHEAIAREESTEFKAGIVSHEVH
RGFLLRER-VLRPAAVKVSNGPGDQNVS-----------STSSEEPVEDTKEDAAV-------------
>TaCGE2B(Traes_2BL_F3EE344D7)
--VAGADPQIVNGEDFPPMNDLIRLYKKAFLDG--NDDV
VSDIEKAITSMEEERSKAASQLESITAEIASGKNKFLRLNADLENFRKQAEKDRAKFTSNIQVELVQNLLPLVDS
FEKTNVEVTLETEKEQKISTSYQGIYKQLVETLKSLGVGVVETVGKPFDPVVHEAIAREESTEFKAGIVSHEVH
RGFLLRER-VLRPAAVKVSTGPGDQIAG-----------ATSSEEPVEDTKEDAAV-------------
>BdCGE2(Bd5g10250)
--VAGADPKIVNGEDFPPMNDLIRLYKKAFLDG--NDDV
VSDIEKAIIGMEQEKSKAASQFESITAEIISGKSKFLRLNADLENFRKQTEKDRAKFTSNIQVELVQSLLPLVDSF
EKANLELTLETDKEQKISTSYQGIYKQLVETLKGLGVGVVETVGKPFDPLVHEAIAREESVQFKAGIVSHEVHR
GFLLRER-VLRPATVKVSTGPGDQNSN-----------ALPTEEPVEDTQEDAVV-------------
>ObCGE2(Ob04g20160)
--AAGADPKIVNGEDFPPMKDLIRLYKKAFLDG--NNEA
LGGIESAIIAMEKERSSSAAQLESITTEITSGKDKFLRINADLENFRKQTEKDRARFTSNIQVEVVQSLLTLADSF
EKVNQEITPETEKEQKISTSYQGIYKQLVETLRSLGVGVVETVGKPFDPSIHEAIAREESHQFKAGIVSHEVRRG
FLLRER-LLRPATVKVSTGSGNQETS-----------SPSSEKPVEDSKEDAAV-------------
>OsJCGE2(LOC_Os04g35180)
--AAAADPKVVNGEDFPPMKDLLRLYKKAFLDG--NDE
ALGGIESAIIAIEKERSNSAAQYESIATEITSGKDKFLRINADLENFRKQTEKERARFTSNIQVDVVQSLLTLVDSF
EKVNQEITPETDKEQTISTSYQGIYKQLVETLRSLGVGVVETVGKPFDPSIHEAIAREESHQFKAGIVSHEVKRG
FLLRER-LLRPATVKVSTGSGTQETS-----------SPSTEKPVEDSKEDAAV-------------
>SbCGE2(XP_002447849)
---VAAGSGVDPKVVNGEDFPPMKDLIQLYRTAFQQG--ND
EVLGEVEKAITAVEKEKSRVASQFESITTEITSGKEKFIRLNADLENFRKQTEKDRAKFTSNMRVQVVQSLLPLV
DSFEKTNLENTPETEKEQKISTSYQGIYKQLVETLRYLGVGVVETVGKPFDPSVHEAISREASMQFKAGIVMHE
VRRGFHLKER-LLRPATVKVSTGSGKQSAS-----------S----------------------------
>ZmCGE2_Chr2(GRMZM2G035948)
---VAAGSGVDPKVVNGEDFPPMKDLIQLYRTAFLEG--NEE
VLGVVEKAITAVEKEKSTIASQFESITTEITSGKEKFIRLNADLENFRKQTEKDRAKLTSNIRVEVVQSLLPLVDS
FEKTNLENTPETEKEQKISASYQGIYKQLVETLRYLGVGVVETVGKPFDPSVHEAISREASMQFKAGIVMHEVR
RGFHLKER-LLRPATVKVSTGSGKQSAS-----------S----------------------------
>SiCGE2(Si010777mg)
---AGAGVDPKVVNGEDFPPMKDLIQLYKTAFLDG--NDE
VLGEVEKAITSVEKEKSRVASQFESVTAEITSGKEKFIRLNADLENFRKQTEKERAKFTSNIQVEVVQSLLPLVD
SFEKTNLENTPETEKEQKISTSYQGIYKQLVETLRYLGVGVVETVGKPFDPSVHEAIAREESSQFKAGIVSHEIR
RGFLLKER-LLRPATVKVSTGSGKQSAS-----------SVEAAKDAAV--------------------
>EsCGE2(XP_006414526)
---AHQASSTDSGEPNSKLPTDVKALIRAYKQAISNG--DET
TVSEIETIFCGIEKEKNRFDNKVLSLSMKIASEKETKIRLQADFDNTRKKLDKDRLSTESNAKVQIMTSLLPLIDS
FEKARQQIKPDTEKEKKIDTSYQGIYRQFVEVLRHLRVKAIATVGKPFDPLLHEAISREESEVVKAGIITEELNR
GFLLGDR-VLRPAKVKVSLGTIKKKTP-----------SAAEEITPSP--------------------
>BrCGE2(asmbl_601napapasmblv1_pasa8)
---AHQASPTNGEETNSKHPPDVKTLIKAYKQALFNG--DQL
TLTEIELFFCEIEKEKNRFDHKVLSLSMKIASEKDTKIRLQADFDNTRKKLDKDRLSTESNAKVQVMKSLLPLID
SFESARQQIRPDTETEKKIDTSYQGIYRQFVEVLRHLRVKAIPTVGKPFDPLLHEAISREKSEAVKVGMITEELTR
GFCLGDR-VLRPAKVKVSLGPIKKKTV-----------SPADETTPSA--------------------
>CruCGE2(XP_006305497)
---ASSPSNSEEANSKYPTDVKSLIRVYKEALFNG--DESSV

VEIERMFCRIEKEKNKMDQKVLSLSMKIASEKEMKIRLTADFENTRKKLDKDRLSTESNAKVQIMRSLLPLIDS
FEKAKLQVSVDTEKEKKIDTSYQGIYRQFVEVLRHLRVAAIATVGKPFDPLLHEAISREESETVKAGIITEELNR
GFLLGDR-VLRPAKVKVSLGPISKKTP-----------SATEEITPSS---------------------
>AlCGE2(XP_002893916)
---ANSKQQADVNTLIRSYKQALFNG--DETSVAQI
ETMFCKIEKEKNKMDQKVLSLSMKIASEKETKIRLQADFDNTRKKLGKDRLSTESNAKVQIMKSLLPIIDSFER
AKLQVRVDTEKEKKIDTSYQGIYRQFVEVLRHLRLSAIATVGKPFDPLLHEAISREESEVVKAGIITEELKRGFV
LGDR-VLRPAKVKVSLGPVKKKTP-----------SPAEEITPSS---------------------
>AtCGE2(NP_849751)
---ANSKQQADVKTLIRSYKQALLNG--DETSVTEI
ETMFCKIEKEKNKMDQKVLSLSMKIASEKEMKIRLQADFDNTRKKLDKDRLSTESNAKVQILKSLLPIIDSFEK
AKLQVRVDTDKEKKIDTSYQGIYRQFVEVLRYLRVSVIATVGKPFDPLLHEAISREESEAVKAGIITEELNKGFV
LGDR-VLRPAKVKVSLGPVNKKTP-----------SAAEEITPSA---------------------
>StCGE2(XP_006366929)
-SSSKAARTSIPPFAQCTHHNFSLNKQCETTKFALSRWNPIIANHTRRSSAKTCLSFQDSAPEASDDEDNLSELEM
SKDVADEKYSSRLKPLMQVYKEAILVG--DVKLISEIEAVISSVDKERDDMSQKVSALSADINSGKEKYIRLQAD
FDNYRKRSENEKLRIRTNAQGEIIESLLPMVDNFERAKRQIKLETEMEKKIDASYQGIYKQFVEIMRSLRVAVVP
TVGKPFDPALHEAIAREESQEFSEGIVIEEFRRGFLLGDR-LLRPAMVKVSSGPGKRVPS-----------SVTQKSPAAT
VGVDES---------------
>FvCGE2(XP_004307232)
---AAARGYVPPVNAKEDN------VQTSGADQQHLPSLRTLLKVYKDAIF
NG--DEQTVAEIEAKIEIVENKQNELVQKVSSMSAEVTSGKEKLIRLQADFDNCRKRFEKERLNVRTDAQGEVIE
SLLPMVDNFERAKQQIKPETEKEKKIDTSYQGIYKQFVEIMRSLRVASVPTLGKPFDPSVHEAIAREESQEFPDG
IVIQEIRRGFLLGGR-LLRPALVKVSIGPGSKKSP-----------VATEKSSGSPATTASVEN-------------
>PpeCGE2(XP_007225704)
--ASESGADQHHLPRLGTLLQVYKEAIFNG--DEETVS
EVEAKIEILENEKNKLVKKVSSSSAEITSGKEKFIRLQADFDNCRKRFEKERLTVRTDAQGEVIESLLPMVDNFE
RAKQYIKPETDKEKKVDASYQGIYKQLVETMKSLHVAVVPTVGKSFDPSLHEAVAREESQEFKEGIIIQEIRRGF
LLEGR-LLRPAMVKVSTGPGSKKAP-----------VATEKSSGLPATAAGVEK-------------
>JcCGE2(KDP32732)
---AEDYAPTTKDKEENHQNGMK------ASEKAADGKTVVGLENLIDIYKAA
VLHG--DERTIADIEARIKIIESENYELVQRVSALSAEIASGKEKYIRLQADFDNFRKRSEKERLNVRSDAQGEVIE
SLLPMVDNFERAKQQLKPETEKEKKIDTSYQGIYKQFVEIMRSLQVAVVATVGKPFDPSLHEAIAREESQEYEE
GIIIQEFRRGFLLGDR-LLRPAMVKVSSGPGRKKAP-----------INAEQSATAAGVDDR----------------
>PtCGE2(XP_002301021)
-------------------------------ASLLSSSSPIPINNIKRRSLKTYLAPEDSAPTTNGKEE------NNEIQEGQKRGPSLKNLM
KIYRQAIFYG--DEKTILDIEAKVATIEKENHEFLQKVSSLSAEITSGKEKYIRLQADFDNFRKRSDKERVNIRSDA
QGEVIESLLPMVDSFERAKQQIQPETEKEKKIDSSYQGIYKQLVDIMRNLQVAAVPTVGKPFDPSLHEAIAREES
QEYKEGIIIQEFRRGFLIGNR-LIRPAMVKVSSGPGNKKSS-----------VGTETRAEQPATAAGMD--------------
>CmCGE2(XP_008445362)
---SPLTRSFAPCLSAHSSVANVNNEEDDG---KAVEKDGYKFDGSSLQTLIEVYR
EAFLDG--DQKTVSEVEARIKIIGREKDELSRKLSNILTEMTSGKEKYIRLQADFDNFRKRSEKEQHNVKNNAQK
EVIESLLPMIDHFEKARQQIVPQTDKEKKIDISYQGIYKQFVETLRSWRVSAVATVGRPFDPSLHEAVAREESQEI
KEGIVIQELRRGFLLGER-LLRPARVKVSKGPGRKSSR-----------TIDGKQQPAAAAGVDEH--------------
>CsCGE2(XP_004150939)
---AMEKDGYKFDGSGLQTLIEVYREAFLDG--DQKTV
SEVEARIKIIGREKDELSRKLMNISTEMTSGKEKYIRLQADFDNFRKRSEKEQHTVKNNAQKEVLESLLPMIDH
FDKARQQIVPQTDKEKKIDISYQGIYKQFVETLRSWRVSAVATVGRPFDPSLHEAVAREESQEIKEGIIIQELRRG
FLLGER-LLRPARVKVSKGPGRKSSR-----------TVDGEQQPAAAAVVDEH--------------
>CaCGE2(XP_004515658)
---SSLNRTELFKSYLASQDSIPTTNDDTEETQNDTR--VKNEDKKPLTSLVVLFEE
YKKSFLNG--NEKTASQIEERIQSKANKKNKLSQKVSSLSADKVSCKEQYLRLQADFDNFRKRCDKERISIQSDA
QLEFIKKLLFMVDHFERVKQQSEAATEKEKKIDASYQSIYKQFVETLRSHHVSVVATVGKPFNPLLHEAVAREE
SDVFKEGIIIKESRRGFLLRDK-VLRPALVKVSLGPGNKKSS-----------VAPAKSLEQ--TAARIDER------------
>MtCGE2(XP_003601139)
---------------------------------PFYASNTILGFPTLNSTSSLFASKDSFPTTNDKTDETR-------NGEDKQDLPSLLVLIKA
YKKAFLNS--DRKTALLIEEIIHSKANERNKLIQKASTLSVDKVSCKEQYLRLQADFDNFRKRCEKERISIQSDAQ
QEFVKKLLLMVDSFERVKQQIEAETEKEKKIDASYQSLYKQFVETLRSHHVSVVATVGKPFNPLLHEAVGREES

EVFKEGIIIKESRRGFMLKDK-VVRPALVKVSLGPGNKKSS-----------VAPTQSLEQPSTAARIDER------------
>GmCGE2(XP_003526604)
--------------CSTPFSSKPFQTLKPQILSFPSRGFPRPLISSVNRRTQLKSSQDFPPTTNDETEETQNDVRGTNDDEDK
KQVPSLMILLEAYKEAFFNG--DQNTVAQIEEGIYSIANRKNKLIQKLSSLSADKAASKKSYLRLQADFDNFRKR
TDKERLNIQSDAQQQVIEKLLLMVDNFERTQQQIKAATEKEKKIGVSYQGIYKQFVEVLRNHNVSVVATVGKP
FNPLQHEAVAREESTEFKKGIIIKESRRGFLLRDR-VLRPALVKVSLGPGNKKSP-----------VSPDKSVEQPSTAAGI
DER------------
>PvCGE2(XP_007136196)
--NDETGETRKDLRGIDGDEDKKPLPSLMVLLEAYKEAFFNG--
DENVVALIEEKIYSELNRKNKLIQKVSSLSADKATSKDRYFRLQADFDNFRKRFDKERLSIQSDAQQELIEKLLL
MVDSFEKTKQHIKAATEKEKKIDVSYQGIYKQFVEILRSHHVSVVATVGKPFNPLQHEAIAREESTEFKKGIIIKE
SRRGFLLRDR-VLRPALVKVSLGPGTKKSP-----------LSPDNSMEQPSTAAGIDER------------
>OsJCGE1(LOC_Os02g39870)
--ADEAAQTATEEDSETGETG---ADDAAAATEETPSVIVTALQSYKEALI
ND--DETKVAEIEDFLFSIEEEKNSLLSKISTLGAELTTERDRILRISADFDNYRKRVEREKLSLMTNVQGEVIESL
LPVLDNFERAKTQIKVETEQETKINDSYQSIYKQFIDILNSLGVEDVETVGKPFDPMLHEAIMREESVEYEEGVI
LQEFRKGFKLGER-LLRPAMVKVSAGPGPEKPV--------YDDPAMVEDSVAPQKVKEAEDDGFDDDNAE----
>BdCGE1(Bd3g48540)
--ADEAAQTATEEDTETEVTGDAVSDDGPVGTEEAPSVLVTALQSYKE
ALMNE--HEAQVAEIESFLLSIENEKNSLMSQITTLDAELTTEKDRILRISADFDNYRKRTEREKLSLMTNVQGEV
VESLLPVLDNFERAKTQIKVETEREAKINDSYQSIYKQLIEILNSLGVEDVETVGKPFDPMLHEAIMREESVEYE
EGVIIQEFRKGFKLGER-LLRPAMVKVSAGPGPEKSE--------DDDPTMVEDNVAPQKADDNEDDGFDDVDAE---
-
>HvCGE1(BAK06504)
----------------------------AQLLTTRRAPGHVASRLRRLGATEADESAQTATQEDTETEVTEDTVADDGSVGTEETPS
VLIIALQSYKEALMND--DEAKIAEIESFLLSIEDEKISLMSKITALDAELTTERDRILRISADFDNYRKRTEREKLS
LMTNVQGEVVESLLPVLDNFERAKTQIKVETEREAKINDSYQGIYKQLVEILNSLGVEDVKTVGKPFDPMLHE
AIMREESVEYEDGVVLQEFRKGFKLGER-LLRPAMVKVSAGPGPEKSG--------DDDTTIGEDSVGPQAVDDGED
DGFDDADAE----
>TaCGE1(AK331943 translate)
------------AASLACSRRPPVRSLRAQLLTTRRAPGHVASRLRRLGATEADESAQTATQEDTETEVTGGTVADDGS
AGTEETPSVLITALQSYKEALMND--DEAKIAEIESFLLTIEDEKNSLMGKITALDAELTTERDRIVRISSDFDNYR
KRTEREKLSLMTNVQGEVVESLLPVLDNFERAKTQIKVETEREAKINDSYQSIYKQLVEILKSLGVEDVETVGK
PFDPMLHEAIMREESVEYEDGVVLQEFRKGFKLGER-LLRPAMVKVSAGPGPEKSG--------DDDTTIGEDSVGP
QAFDDGEDDGFDDADAE----
>SiCGE1(Si017771mg)
---ATEADEAAQTATQEDSETEVTGDSAADDGAGSTDETPSIIVTALQSYKE
ALIND--DEAKAAEIEAFLLSIEDEKNSFMNKITVLDAELATQRERILRISADFDNFRKRTENEKLNMMSNVQGE
LIENFLPVLDNFERAKSQIKVETEGEEKINNSYQSIYKQFIEILNSLGVEDVETVGKPFDPMLHEAIMREESTEYE
EGIILQEFRKGFKLGER-LLRPAMVKVSAGPGPEKSG-------DDEDPTEVEDSVAPLKVEDAEDDDGDAE-------
>SbCGE1(XP_002452436)
-----------------CSRRAPARPLRAWLLPTPHVFCRDGARFRRLAATEADEAAQTATQEDSETEVTGDSAADDGAGS
TDETPSIIVTTLQSYREALIND--DEAKAAEIESFLLSIEDEKNSLLNKITALNAELATQRERILRISADFDNFRKRT
ENEKLNMMENVQGELIESFLPVLDNFERAKMQIKVETEGEEKINNSYQSIYKQFIEILNSLGVEDVETVGKPFD
PMLHEAIMREDSSEYEEGIILQEFRKGFKLGER-LLRPAMVKVSAGPGPEVSAGPGPEVSRDDDPTVVEDSVAP
QKIEDVEDDGVDGDAE-----
>ZmCGE1_Chr5(GRMZM2G036590)
------------------------QLRAWPLPTPHVFGRSGGRFRRLSVTEAEEAAQTAAQEDPETEVTGESAADDSAGSTDET
PSIIVTTLQSYRVALIND--DDAKAAEIESFLLSIEDEKNSLLNKITALDVELATQRERILRISADFDNFRKRTENEK
LNMMENVQGELIESFLPVLDNFERAKMQIKVETEGEEKINNSYQSINKQFIEILNSLGVEDVETVGKPFDPMLH
EAIMREESSEYEEGIILQEFRKGFKLGER-LLRPAMVKVSAGPGPENSG--------DDDPTVVEDSVAPQKVEDVEE
DGFDDGDAE----
>ZmCGE1_Chr4(GRMZM2G044684)
-----------------CSRRAPVRPLRAWPLPTPHVFCLAGGRLRRLGATEADEAAQTATQEDLETEVTGDSAADDSAGS
TDETPSIIVTTLQSYREALIND--DEAKAAEIESFLLSIEDEKNSLLNKITALDAELATQRERILRISADFDNFRKRT
ENEKLNMMENVQGELIESFLPVLDNFERAKVQIKVETEGEEKINNSYQSIYKQFIEILNSLGVEDVETVGKPFDP
MLHEAIMREESSEFEEGIILQEFRKGFKLGER-LLRPAMVKVSAGPGPEKFG--------DDDPTAVEGSVAPQKVDE
VEDDGFGAGDAE----

>AtrCGE1(XP_011629290)
---------------------------------------AFSSSGEVTETEELEGAQTLQETTEDEAPVEAETVKPDSIDGEELPVSAVKASLE
SYREALANN--DESTLVEIEKFLQSIEVEKNSLSNKVAALSEELSSERDRVLRISADFDNFRKRTERERLSLVSNAQ
GEVIENLLPILDNFERAKAQIKVETEGEEKINNSYQSIYKQFVEIMVSLGVVGVDTVGNPFDPLLHEAIMREDST
EFEESIILQEFRKGFKLGDR-LLRPSMVKVSAGPGPAKPT--------DEVDSSASKEPAEEVDSSASKEETEEEPASE--
-
>StCGE1(XP_006339226)
CGQRQTPLLPRRCRIHHSANSIVQFQHRSVEKFVIFASNEDVAEAAETETLEPQQVDESEQEENLDGAASEEAS
DEDDNAAAEETLSVVATSLQLYRDALANN--DDSKVAEIEISLKSIEDEKIELQTKVASLTEELSSERDRVLRISAD
FDNFRKRTDRERASLVTNAQGEVVEKLLSVMDNFERAKMQIKVATEGEEKISNSYQSISKQFMEILGSLGVEPV
ETVGKPFDPLLHEAIMREDSSEFEEGVVLEEYRKGFKLGDR-LLRPSMVKVSAGPGPAKPE--------TTEPKEEEH
NETDEKSEEGTAETPGDEGTGEGGN
>AlCGE1(XP_002873846)
---ASGEAETTETEVESNKPEVQETDGAVDVENENAGAEEVEAEEEEAAVVTALL
NSYKEALADN--NEGKIAEIEASLKSIEDEKNLLADKVASLSNELSVERDRLIRISADFDNFRKRTERERLNLVSN
AQGEVVENLLAVLDNFERAKSQIKVETEGEEKVTNSYQSIYKQFVEILGSLGVIHVETVGKQFDPMLHEAIMR
EDSAEYEEGIVLEEYRKGFLLGER-LLRPSMVKVSAGPGPEKPR--------EAEVEETTAQGSSEEEASSS-------------
-
>AtCGE1(NP_850840)
---ASGEAETTETEVESNEPEVQETDGAVDVENENASAEEGEAEEEEAAVITALLK
SYKEALADN--NEGKIAEIEASLKSIEDEKFLLADKVASLSNELSVERDRLIRISADFDNFRKRTERERLNLVSNA
QGEVVENLLAVLDNFERAKSQIKVETEGEEKVTNSYQSIYKQFVEILGSLGVIHVETVGKQFDPMLHEAIMRE
DSAEYEEGIVLEEYRKGFLLGER-LLRPSMVKVSAGPGPEKPL--------EAEGEEATAQGSAEEESSSS--------------
>CruCGE1(XP_006288204)
---ASGEAETTETEVESNEPEVQETDGAVGVESENVVAEEGEAEEEDAAVITALLN
SYKEALAEN--NEGKIAEIEASLKSVEDEKFLLADKVASLSSELSVERDRLIRISADFDNFRKRTERERLNLVSNA
QGEVVENLLAVLDNFERAKSQIKVETEGEEKVTNSYQSIYKQFVEILGSLGVTHVETVGKQFDPMLHEAIMRE
DSAEYEEGIVLEEYRKGFVLGER-LLRPSMVKVSAGPGPEKPG--------EAEGEEASAQGNSEEEASSS--------------
>EsCGE1(XP_006400314)
---ASGEAETTETEVESSEPEVQETD-------GAVGVEGENAEEEEAAVTIALLNSYK
EALAEN--NEEKIAEIEASLKSIEDEKFQLEEKVASLTNEMSVERDRLIRISADFDNFRKRTERERLNLVSNAQGQ
VVENLLAVLDNFERAKSQIKVETEGEEKVTNSYQSIYKQFVEILGSLGVVTVETVGRQFDPMLHEAIMREDSA
EYEEGIVLEEYRKGFLLGER-LLRPSMVKVSAGPGPEKPR--------EAEGEEGTAEGSTDEEEAEASSS-----------
>BrCGE1_A02(asmbl_739napapasmblv1_pasa1)
---------------------ASLRSVSGGYPLRLLNFAPFASGESETTETEVESNEPQVQETDGAV----GVESEDTSTEEEGEAA
VTIALLSSYKEALAEN--NEEKIAEIEASLKSIEDEKFQLEDKVASLTNELSVERDRLIRISADFDNFRKRTERERL
NLVSNAQGEVVESLLAVLDNFERAKSQIKVETEGEEKVTNSYQSIYKQFVEILGSLGVVTVETVGKQFDPMLH
EAIMREDSAEYEEGIILEEYRKGFLLGER-LLRPSMVKVSAGPGPEKTQ--------EA--------------------------------
>BrCGE1_A10(asmbl_1432napapasmblv1_pasa10)
-------------------------SVSGGYPLRLLNFVPFASGEAETTETEVESSEPEVQETD-------GAEGEN-AGAEEEEAAVTI
ALLSSYKEALAEN--NEEKIAEIEASLQSIEDAKFQLQEKVATLSNELSVERDRLIRISADFDNFRKRNERERLNLV
SNAQGEVVENLLAVLDNFERAKSQIKVETEGEERVTNSYQSIYKQFVEILGSLGVVTVETVGKQFDPMLHEAI
MREDSAEYEEGIVLEEYRKGFLLGER-LLRPSMVKVSAGPGPEKAG--------EGEGEEAIAQGSAEGAEATSS------
----
>FvCGE1(XP_004287210)
-----------------FSHFPSLGFVPFASQQQQGETDTTDTLDAQDLEEEDSLDGAVSVEDSTSDAEESGIS-DDGEADDK
KPASAVIASLQLYKEALASN--DESKVAEIESFLKSIEDEKIGLEMKVASLSEELSAEKVRILRISADFDNFRKRTD
RERVSLVTNAQGEVVESLLGVLDNFERAKSQIKIETEGEEKINKSYQSIYKQFGEILSSLGVLPVETVGKPFDPV
FHEAIMREDSSEFEEGIILDEFRKGFKLGDR-LLRPAMVKVSAGPGPAKPE--------QQVAPSVEDDAIETTEDGAK
AESA----------
>PpeCGE1(XP_007202333)
------------------------------ASQGETETTETVEEVRQPEEIEDSSDGAVSVEDSTSDGEESGTSNDDEGDAEEKPVSAII
ASLQLYKEALASN--DESKVAEIESFLKYIEDEKISLEKKVASLSEELSAEKVRILRISADFENFRKRTDRERISLVT
NAQGEVVESLLPVVDNFERAKTQIKVETEGEEKINNSYQSIYKQFGEILSSLGVVPVETVGKPFDPLLHEAIMR
EDSTEFEEGVIIDEFRKGFKLGDR-LLRPSMVKVSAGPGPAKPD--------QQVPPSEEQDASETTKEGSTETESA----
-----
>JcCGE1(KDP39624)
--------------------SSHRFIKFVPFATQGETETTETEETIQEPEIQDSSDGAVGVEGSADELEDSSSS--EEADDTKEAPS

VILASLQSYKEALASN--DESKIVEIEAFLKSVEDEKINLEQKVVSLSQDLSTEKDRILRISADFDNFRKRTERERL
SLLTNAQGEVVESLLPVLDNFERAKSQIKLETEGEEKINNSYQSIYKQFVEILGSLGVVPVETIGKPFDPLLQEAI
MREDSTEFEEGIILDEFRKGFKLGDR-LLRPAMVKVSAGPGPAKAE--------EAGSPGEAETASETNEESAAEPEPE
SQPEST---
>PtCGE1(XP_002319738)
--DSSDGAVEVEDAASSEEVASSEEVADAEDTSSVVMASLRSYKEAL
ASN--DESIIAEIEAFLKSVEDEKIDNERKVASLTEELSIEKERVLRISADFDNFRKRTERERLSLVTNAQGEVVEN
LLSVLDNFERAKTQIKTATEGEEKINNSYQNIYKQFMEILVSLGVVPVETIGKPFDPMLHEAIMREDSDAFEEGT
VLEEYRKGFKLGDR-LLRPSMVKVSAGPGPVKPE--------QVEESQEEAEATSGTSEGGSTEEESA--------
>CmCGE1(XP_008455767)
---DSEAEDSSVSYTGVEDATSDNDISDDS---EVNAEDSTQSVIVAALQSYKQ
ALSDN--NGAQIVEIESFLKSIEDEKLAVERKLNSLIEELSVEKDRVLRISADFDNFRKRTERERLSLVKNAQGEV
VETLLGVLDNFERARAQIKVETEGEEKINQSYQSIYKQFTEILGSLGVVPVETIGKPFDPLLHEAIMREDSTEFE
DGIILDEFRKGFLLGDR-LLRPSMVKVSAGPGPEKSD--------ETAPAEELDSSEEFANSESESS------------
>CsCGE1(XP_004157987)
---DSEAEDSSVSYTGVEDATSDNDISDDS---EVNTEDSTQSVIVAALQSYKQ
ALADN--DGAQMVEIESFLKSIEDEKLAVERKLSSLIEELSVEKDRVLRISADFDNFRKRTERERLSLVKNAQGEV
VETLLGVLDNFERARAQIKVETEGEEKINQSYQSIYKQFTEILGSLGVVPVETIGKPFDPLLHEAIMREDSTEFEE
GIILDEFRKGFLLGDR-LLRPSMVKVSAGPGPEKSD--------ETAPAEKLDSSEEFANSESESS------------
>CaCGE1(XP_004493255)
--------VSVTFSRRNPSPLSILRFPSIPSLRFVKLVPFAFDGDSEAPQVQEP--PEVQDSSDGAIGVEDSAGDDEFSDA
GEIPSSPLIVLLQSYKEALANN--DEVKVAELESSLKSIDDEIVGLEGKIASLSEELSIEKDRKLRIGADFDNYRKR
TERDRLSLVTNAQGEVVESLLPVLDNFERAKAQIKVETEGEEKINNSYQSIYKQFIEILNSLGVEPVETVGKPFD
PMLHEAIMREDSAEFEDGIIIQEFRKGFQLGDR-LLRPSMVKVSAGPGPAKPE--------QEVSQEEQVTNEISQDSK
ENDGSTETESV-----
>MtCGE1(XP_003624768)
--------VSVASSRRRPSPLKSHRFSSIPTLRFAKLVPFAFDGDTEAPQVQDSPEVQVLDSTDGAADVEESTGDDEVS
DAGEIPASPLIVLLQSYKEALANN--DSVKVAELESSLKSIDDEIVGLEGKIASLSEELSIEKDRKLRIGADFDNYR
KRTDRDRLSLVTNAQGEVVESLLPVLDNFERAKAQIKVETEGEEKINNSYQSIYKQFIEILNSLGVEPVDTVGNP
FDPMLHEAIMREDSDEFEDGIILQEFRKGFKLGDR-LLRPSMVKVSAGPGPAKPE--------QEVPQEEEVTNETSQ
DSKDNDGNTETESA-----
>GmCGE1(XP_003520909)
---APQVQEP-EVQVLDPSDGAVGVNDSASDNEVSDADETFASPFLLLLQSY
KEALANN--DEVKIAELESSLKSVEDEKIELEVKIASLSEELSVEKDRILRISADFDNFRKRTERDRLSLVTNAQG
EVVESLLPVLDNFDRAKTQIKVETEGEEKINNSYQSIYKQFIEILNSLGVEPVETVGTPFDPLLHEAIMREDSDEF
EDGIIIQEFRKGFKLGER-LLRPSMVKVSAGPGPAKPE--------QEAPQEEHGNTEISEDSKQNEGSTETES------
>PvCGE1(XP_007161911)
---APQVQEP-EVQVLDPLDGAVDVKDNASDNEGSDADEASASPFVLLLQSY
KEALASN--DEVKITELESSLKSIEDEQKDLEGKIAALSEELSIEKVRILRISADFDNFRKRTERDRLSLVTNAQGE
VVESLLPVLDNFERAKTQIKVETEGEEKVNNSYQSIYKQFNEILTSLGVEPVETIGTPFDPLLHEAIMREDSAEFE
DGIIIQEFRKGFKLGDR-LLRPSMVKVSAGPGPAKPE--------QEAPQEDKVNTEISEDSKEIEGGTEADSA-----
